# Genomic and Immunogenic Protein Diversity of *Erysipelothrix rhusiopathiae* Isolated From Pigs in Great Britain: Implications for Vaccine Protection

**DOI:** 10.3389/fmicb.2020.00418

**Published:** 2020-03-13

**Authors:** Taya L. Forde, Nichith Kollanandi Ratheesh, William T. Harvey, Jill R. Thomson, Susanna Williamson, Roman Biek, Tanja Opriessnig

**Affiliations:** ^1^Institute of Biodiversity, Animal Health & Comparative Medicine, University of Glasgow, Glasgow, United Kingdom; ^2^Disease Surveillance Centre, SAC Veterinary Services, Scotland’s Rural College, Edinburgh, United Kingdom; ^3^Surveillance Intelligence Unit, Animal and Plant Health Agency, Bury St Edmunds, United Kingdom; ^4^The Roslin Institute, The University of Edinburgh, Midlothian, United Kingdom

**Keywords:** antigen, *Erysipelothrix rhusiopathiae*, genomics, Great Britain, pigs (*Sus domesticus*), vaccine, whole genome sequencing

## Abstract

Erysipelas, caused by the bacterium *Erysipelothrix rhusiopathiae*, is re-emerging in swine and poultry production systems worldwide. While the global genomic diversity of this species has been characterized, how much of this genomic and functional diversity is maintained at smaller scales is unclear. Specifically, while several key immunogenic surface proteins have been identified for *E. rhusiopathiae*, little is known about their presence among field strains and their divergence from vaccines, which could result in vaccine failure. Here, a comparative genomics approach was taken to determine the diversity of *E. rhusiopathiae* strains in pigs in Great Britain over nearly three decades, as well as to assess the field strains’ divergence from the vaccine strain most commonly used in British pigs. In addition, the presence/absence and variability of 13 previously described immunogenic surface proteins was determined, including SpaA which is considered a key immunogen. We found a high diversity of *E. rhusiopathiae* strains in British pigs, similar to the situation described in European poultry but in contrast to swine production systems in Asia. Of the four clades of *E. rhusiopathiae* found globally, three were represented among British pig isolates, with Clade 2 being the most common. All British pig isolates had one amino acid difference in the immunoprotective domain of the SpaA protein compared to the vaccine strain. However, we were able to confirm using *in silico* structural protein analyses that this difference is unlikely to compromise vaccine protection. Of 12 other known immunogenic surface proteins of *E. rhusiopathiae* examined, 11 were found to be present in all British pig isolates and the vaccine strain, but with highly variable degrees of conservation at the amino acid sequence level, ranging from 0.3 to 27% variant positions. Moreover, the phylogenetic incongruence of these proteins suggests that horizontal transfer of genes encoding for antigens is commonplace for this bacterium. We hypothesize that the sequence variants in these proteins could be responsible for differences in the efficacy of the immune response. Our results provide the necessary basis for testing this hypothesis through *in vitro* and *in vivo* studies.

## Introduction

*Erysipelothrix rhusiopathiae*, the causative agent of erysipelas, remains a persistent challenge for swine and poultry production systems worldwide. Although well-controlled for decades through vaccination, erysipelas is re-emerging in several European and Asian countries (Eriksson et al., 2013; Kwok et al., 2014; Janßen et al., 2015; Ogawa et al., 2017). *E. rhusiopathiae* was also recently implicated in large-scale mortalities and population declines in muskoxen and other wild ungulates in North America (Kutz et al., 2015; Forde T.L. et al., 2016). While *Erysipelothrix* spp. infection can impact all pig production stages and is a common and significant cause of carcass condemnation, its true economic burden is largely unknown. The *E. rhusiopathiae* genome is comprised of a single chromosome of about 1.8 million base pairs (MB), with an average GC content of 36.5% (Ogawa et al., 2011; Kwok et al., 2014; Tang et al., 2016). Comparative genomic analysis of a diverse collection of *E. rhusiopathiae* isolates from a wide range of host species recently led to a better understanding of the global diversity and population structure of this species (Forde T. et al., 2016), including its propensity for homologous recombination. Strains can be broadly divided into Clades 1, 2, and 3, as well as a clade phylogenetically “intermediate” to Clades 2 and 3. The genomic diversity of *E. rhusiopathiae* at smaller (e.g. national) scales has only begun to be explored. However, studies conducted to date illustrate that this may range from highly related clonal strains (e.g. as seen in pigs in Japan; Ogawa et al., 2017), to phylogenetically diverse strains, as observed in poultry in Germany (Janßen et al., 2015). The degree of genomic diversity present could affect the ability of vaccines to provide protection against the full spectrum of circulating field strains.

**FIGURE 1:**
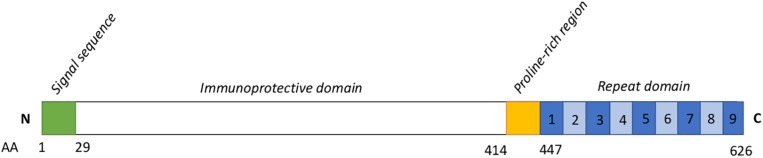
Schematic representation of the SpaA protein sequence. Based on To and Nagai, 2007. Numbers below indicate amino acid (AA) positions. Numbers within the blue boxes represent the typical number of repeat units. The signal sequence (AA 1-29) is thought to be associated with the secretion mechanism for this protein. The immunoprotective domain (AA 30-413) is the component conferring immunogenicity, and upon which typing schemes for SpaA have been developed. The role of the proline-rich region (415–447) has not been well described. Seven to 13 repeats of a 20-AA sequence containing a GW module make up the C-terminal domain of SpaA; these have a role in binding the SpaA protein to the bacterial cell surface.

An important component of *E. rhusiopathiae* diversity that has remained relatively unstudied is that of its surface proteins. Because of their high propensity for host-pathogen interactions, surface proteins often play an important role in virulence mechanisms, as well as in eliciting a host immune response, thereby representing potential vaccine candidates (Gamberini et al., 2005). One of the most critical immunogens for *E. rhusiopathiae* identified to date is the surface protective antigen (Spa)A protein (Makino et al., 1998; Imada et al., 1999; Shimoji et al., 1999). Of the three different Spa types (A, B, and C) that have been found in *Erysipelothrix* spp. (To and Nagai, 2007), SpaA is by far the most widely prevalent Spa type in *E. rhusiopathiae.* In a global collection of *E. rhusiopathiae* isolates examined from various host species, the *spaA* gene was found in more than 90% of isolates (79/86), including all those from pigs and poultry (Forde T. et al., 2016). This corroborates several international studies where the *spaA* gene was the exclusive Spa type found, including from pig outbreaks in Japan (*n* = 83) (To et al., 2012), isolates from pigs in Australia (*n* = 44) (Eamens et al., 2006), and a large study of 165 predominantly poultry isolates from Germany (Janßen et al., 2015). It has been shown that the N-terminal immunoprotective domain of this surface protein – also referred to as the hypervariable domain – is the component responsible for eliciting protective immunity (Figure 1; Imada et al., 1999; Shimoji et al., 1999). SpaA also plays an important role in pathogenesis by increasing resistance to phagocytosis and promoting endothelial adherence (Harada et al., 2014; Borrathybay et al., 2015; Zhu et al., 2017a, b). Several variants (groups) of the SpaA protein have already been described, based on amino acid differences within the immunoprotective domain (Uchiyama et al., 2014; Janßen et al., 2015; Ogawa et al., 2017). While tests have been conducted on both mice and pigs to assess cross-protection among different Spa types using strains representing multiple serotypes (To et al., 2010), it remains unclear whether amino acid variants within the SpaA protein can result in differences in protection. Moreover, the genetic sequence of *spaA* of *E. rhusiopathiae* strains used in vaccines has not yet been characterized.

Additional surface proteins that have a demonstrated role in immunogenicity of *E. rhusiopathiae* include rhusiopathiae surface protein A (RspA) (Shimoji et al., 2003), choline binding protein B (cbpB) (Shi et al., 2013; Zhu et al., 2018) and Glyceraldehyde 3-phosphate dehydrogenase (GAPDH) (Zhu et al., 2018); these proteins have all elicited protection during challenge studies in mice, and the latter two also in pigs. An additional eight putative surface proteins were recently found to give rise to varying degrees of protective immunity in mice (Hu et al., 2017; Table 1). As with SpaA variants, the exact role and the extant diversity of these different surface proteins among *E. rhusiopathiae* strains have yet to be determined.

**TABLE 1:** Surface proteins of *Erysipelothrix rhusiopathiae* with a suspected role in immunogenicity.

			**Locus tag**		**Nucleotide**	**Amino acid**	
	**Gene**	**Name/Description**	**(former)**	**Positions^a^**	**length**	**length**	**References**
1	*spaA*^b^	Choline-binding protein	ERH_RS00500 (ERH_0094)	112,931–114,811	1881	626	Imada et al., 1999; Shimoji et al., 1999
2	*rspA*	Rhusiopathiae surface protein A; B-type domain-containing protein; LPXTG-motif cell wall anchor	ERH_RS03410 (ERH_0668)	701,546–707,524	5979	1992	Shimoji et al., 2003
3	*CbpB*	Choline-binding protein	ERH_RS03905 (ERH_0768)	813,020– 814,840	1821	606	Shi et al., 2013; Zhu et al., 2018
4	GAPDH (*gapA*)	type I glyceraldehyde-3-phosphate dehydrogenase	ERH_RS07885 (ERH_1534)	1,618,401–1,619,405 (rev)	1005	334	Shi et al., 2013; Zhu et al., 2018
5	*Da* (*dap2*)	Dipeptidyl aminopeptidase; S9 family peptidase	ERH_RS00295 (ERH_0059)	62355–64328 (rev)	1974	657	Hu et al., 2017
6	*CbpA*	Collagen-binding protein; cell wall anchor	ERH_RS00405 (ERH_0075)	89263–94584	5322	1773	Hu et al., 2017
7	*Plp*	Pectin lyase fold-containing protein	ERH_RS00860 (ERH_0165)	196,260–200,819	4560	1519	Hu et al., 2017
8	*Atsp*	ABC transporter, extracellular solute-binding protein	ERH_RS01215 (ERH_0228)	262,816–264,261	1446	481	Hu et al., 2017
9	*Neu* (*nanH*)	Neuraminidase; LPXTG-motif cell wall anchor domain protein	ERH_RS01540 (ERH_0299)	331,513–335,127 (rev)	3615	1204	Hu et al., 2017
10	*Bga*	Beta-galactosidase; DUF4982 domain-containing protein	ERH_RS03965 (ERH_0780)	825,779–832,054	6276	2091	Hu et al., 2017
11	*Bml*	Basic membrane lipoprotein; BMP family ABC transporter substrate-binding protein	ERH_RS05080 (ERH_1005)	1,061,289–1,062,380 (rev)	1092	363	Hu et al., 2017
12	*CwpA*	LPXTG-motif cell wall anchor domain protein	ERH_RS07320 (ERH_1428)	1,500,578–1,506,982 (rev)	6405	2134	Hu et al., 2017
13	*CwpB*	LPXTG-motif cell wall anchor domain protein	ERH_RS07450 (ERH_1454)	1,537,616–1,540,657 (rev)	3042	1013	Hu et al., 2017

*All listed proteins have experimentally been shown to be involved in eliciting a host immune response. ^*a*^Positions are in relationship with reference strain Fujisawa (NC_015601). ^*b*^*spaB* and *spaC* sequences, with a role in immunogenicity of *Erysipelothrix* spp., were not found in any of the isolates in this study.*

The objectives of this study were to (1) characterize the overall genomic diversity of *E. rhusiopathiae* in British pigs and assess the amount of genetic divergence between field strains and a commonly used vaccine strain in the United Kingdom (UK), and (2) to quantify the amount of genetic diversity in Spa and other immunogenic surface proteins.

## Materials and Methods

### Bacterial Isolates and Vaccine Strain

Archived *E. rhusiopathiae* isolates from clinical pig erysipelas cases from across England and Wales were provided by the UK Animal and Plant Health Agency (APHA; Supplementary Table S1). A total of 48 isolates were initially selected to uniformly represent as a wide a time frame as possible; dates of isolation ranged from 1987 to 2014. Isolates were also selected for geographic representation, including isolates submitted to 10 different regional Veterinary Investigation Centers (Supplementary File S1, Supplementary Figure S1). To minimize the inclusion of epidemiologically linked isolates (i.e. stemming from a common outbreak), efforts were made to select isolates from different years when originating from the same investigation center. Isolates were serotyped prior to this study using previously described methods (Opriessnig et al., 2013); those selected for inclusion in this study aimed to represent a mix of serotypes 1a, 1b and 2, which encompass 90% of the isolates recovered from clinical erysipelas in British pigs (McNeil et al., 2017). DNA was extracted from isolates regrown on blood agar plates using the DNeasy Blood & Tissue kit (Qiagen). With the same kit, DNA was also extracted directly from one of the most commonly used inactivated *E. rhusiopathiae* vaccines in the UK (referred to hereafter as the vaccine strain; see Commercial Products Used). We also endeavored to perform DNA extraction from a second commercial vaccine, however, this vaccine did not test positive by *E. rhusiopathiae* probe-based qPCR, and DNA of sufficient quantity (as measured by Qubit) could not be extracted despite multiple attempts.

### Library Preparation, Sequencing and Assembly

Library preparation, sequencing and *de novo* assembly were performed by MicrobesNG (Birmingham, UK). Libraries were prepared using the Nextera XT v2 kit and sequenced on the Illumina HiSeq2500 platform, generating 250 base pair paired-end reads. Reads were trimmed using Trimmomatic (Bolger et al., 2014), and assembled *de novo* using SPAdes v. 3.7.0 (Bankevich et al., 2012) with default settings. Assembly quality metrics (i.e. number of contigs, N50) were obtained using QUAST (Gurevich et al., 2013). Three of the isolates submitted had poor sequencing quality indicative of contamination (i.e. unexpected total length and GC content) and were excluded from further analyses, resulting in 45 whole genome *E. rhusiopathiae* sequences (WGS) from British pigs. Since key questions in this study were related to surface antigens and their relationship with a current vaccine strain, the vaccine extract was submitted for sequencing twice on two independent runs for variant confirmation.

### Population Structure

To place the newly sequenced isolates (i.e. the vaccine strain and the 45 British pig isolates from which high quality sequence data were obtained) within the broader global population structure of *E. rhusiopathiae*, a phylogenetic tree was estimated that included an additional 75 isolates from Clades 2, 3 and the intermediate clade (Forde T. et al., 2016) (Supplementary Table S1). The Fujisawa reference genome (NC_015601.1), the first complete high-quality genome for *E. rhusiopathiae* (Ogawa et al., 2011), was also included; this is a virulent serotype 1a strain isolated from a pig in Japan prior to 1985. Serotype was already known for 10/75 of the global isolates. *In silico* serotype testing was also done to detect isolates of serotypes 1a, 1b, 2, and 5. In brief, BLAST searches of all *de novo* assemblies were done using primer pairs described for each serotype (Shiraiwa et al., 2018). A BLAST search was considered positive for a given serotype if there were matches for both forward and reverse primers across their full length with a maximum of one SNP difference, and that yielded the expected amplicon length (i.e. distance between primers). The phylogeny – based on core single nucleotide polymorphisms (SNPs) – was built using Nullarbor^1^ (Seemann et al.) implemented through the CLIMB computing platform for microbial genomics (Connor et al., 2016). The Nullarbor pipeline uses the program Freebayes v1.1.0 for variant calling across all isolates with respect to a reference genome. It subsequently builds a core SNP alignment using Snippy v3.2, where “core sites” are genomic positions that are present in all the included isolates. This core alignment comprised 3490 SNPs. FastTree v2.1.10 was then called within Nullarbor to infer the phylogeny using maximum-likelihood based on the GTR + G4 model of substitution.

### Variability of Spa and Other Surface Protein Sequences

Spa genes were searched for in all newly sequenced isolates by performing BLASTn searches using a custom database of *spaA*, *spaB*, and *spaC* nucleotide sequences from different serotypes available on GenBank (Supplementary Tables S1, S2), implemented within Geneious v. 11.0.5 (Kearse et al., 2012). A BLAST hit was considered positive for a particular Spa type if it had greater than 95% pairwise identity; in practice, homologous Spa types had ∼98% pairwise identity, while heterologous types generally had ∼90% identity or lower. Nucleotide sequences of the *spaA* gene based on the BLAST hits were extracted from all *de novo* assemblies (*n* = 121). *spaA* sequences were similarly extracted from seven whole genome sequences of *E. rhusiopathiae* available on GenBank: Fujisawa, SY1027 (NC_021354.1), GXBY-1 (NZ_CP014861.1), WH13013 (NZ_CP017116.1), NCTC7999 (NZ_UFYF01000001.1), NCTC8163 (NZ_LR134439.1), and ML101 (NZ_CP029804.1). Finally, to further explore the variability of this surface protein, an additional 215 publicly available *spaA* nucleotide sequences were downloaded (Supplementary Table S2), resulting in a total of 343 *spaA* sequences. Translations were performed either using the transeq program from EMBOSS (Rice et al., 2000), or within Geneious, using translation Table 11 for bacteria. Amino acid sequences were aligned using MUSCLE (Edgar, 2004), implemented within Geneious, and any amino acid variants were recorded in comparison with the Fujisawa reference sequence. A phylogenetic tree was estimated from the protein alignment using a Neighbor-Joining method implemented in Geneious Tree Builder, using the Jukes-Cantor model.

The amino acid variability within 12 additional surface proteins that have been shown to play a role in *E. rhusiopathiae* immunogenicity was also examined (Table 1). A custom BLAST database of the nucleotide sequences of these genes was created based on the sequences from the Fujisawa reference genome within the program Geneious. Each *de novo* assembly from British pig isolates and the vaccine strain was queried to identify and extract the homologous gene sequences, which were then translated to amino acid sequences and aligned within Geneious along with the Fujisawa reference sequence. Phylogenies for each protein were estimated as described for SpaA. Variants of these different surface proteins were identified based on clustering observed in mid-point rooted phylogenies (Supplementary File S2).

### Conformation of SpaA Protein Variants

To assess the potential structural impact of SpaA protein variants identified, the 3-D protein structure of a typical group 1 SpaA (Figure 4) was initially modeled using the I-TASSER platform (Roy et al., 2010). I-TASSER uses LOMETS, a meta-threading server that combines several threading programs to detect structural templates from the Protein Data Bank (PDB) using threading or fold recognition. The threading-aligned regions of these templates provide the building blocks and spatial restraints for the prediction of the target protein, with unaligned areas predicted *ab initio*. Full-length structural models were predicted by I-TASSER, and the best structural model – identified through comparison of C-scores – was used for further analysis. C-scores are typically bounded between −5 and 2 with higher values indicating greater confidence in the predicted model and −1.5 acting as a useful threshold above which more than 90% of predictions are correct (Roy et al., 2010). To assess the structural similarity of the SpaA structural model to existing PDB structures, template modeling score (TM-score) and root-mean-square deviation (RMSD) were calculated across aligned Cα atoms using TM-align v20170708 (Zhang and Skolnick, 2005). TM-scores are bounded between 0 and 1 where 1 indicates perfect structural alignment, scores above 0.5 assume roughly the same fold while scores below 0.2 correspond to randomly chosen unrelated proteins (Zhang and Skolnick, 2005). Mutations were introduced using the PyMOL molecular graphics system (Schrödinger^2^), where the most common rotamer was selected and energy minimization was performed in the locality of the mutated site.

Epitope scores were predicted per-residue based on amino acid identity, surface exposure and side chain orientation using BEpro (Sweredoski and Baldi, 2008). Amino acid diversity was calculated using an alignment of all available *E. rhusiopathiae* SpaA protein sequences (*n* = 343) (including residues 1–447 of the N-terminal region). Diversity at each amino acid position was calculated as the true diversity at *q* = 2 (Inverse Simpson index), whereby 1 indicates a fully conserved position and numbers greater than 1 indicate increasing diversity. Amino acid diversity was visualized on the structural model using PyMOL.

## Results

### Sequencing, Population Structure and Serotype

**FIGURE 2:**
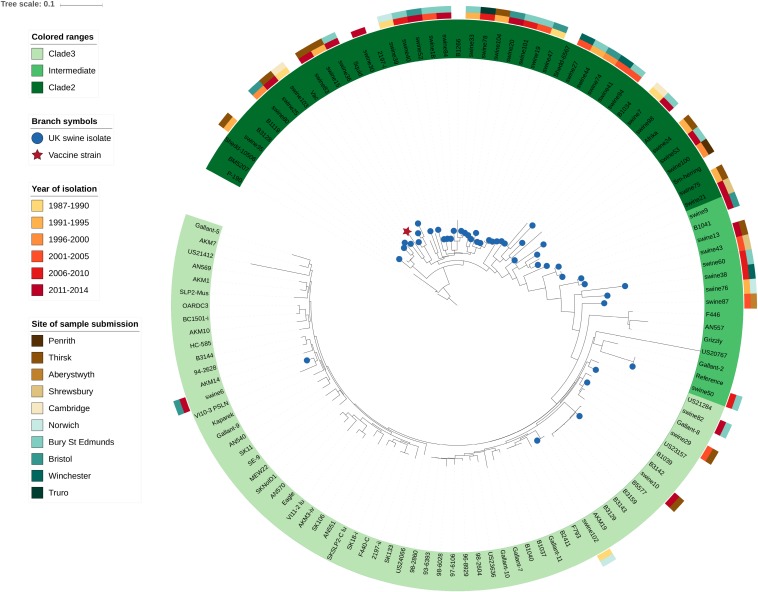
Diversity of *Erysipelothrix rhusiopathiae* in British pigs as shown within a global phylogenetic context. This maximum likelihood phylogeny is based on core single nucleotide polymorphisms (SNPs) identified through the Nullarbor pipeline. British pig isolates (*n* = 45) are represented by blue circles at branch tips, while the vaccine strain is represented by a red star. Branches with no symbol are isolates from a broad global collection (*n* = 75) from various host species and geographic locations representing Clades 2, 3 and the intermediate clade. An isolate from Clade 1 was used to determine the appropriate rooting position (not shown). The Fujisawa strain was used as the reference genome. Spatio-temporal diversity of the British pig isolates is illustrated by color bars to the outside of the phylogeny, with the inner color representing the year range of isolation, and the outer color bar representing the veterinary center to which the isolate was submitted.

**FIGURE 3:**
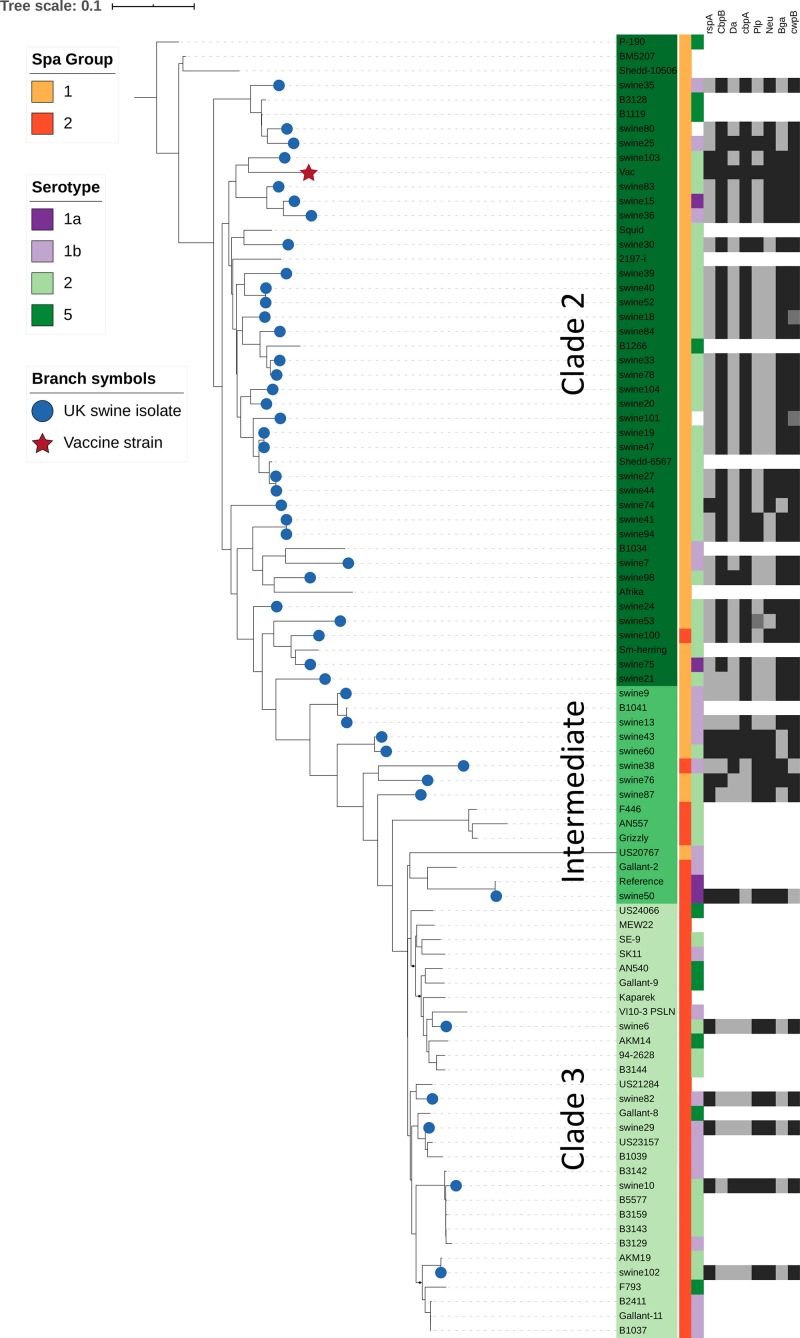
Relationship of SpaA group, serotype, and immunogenic surface proteins to phylogenetic population structure. This phylogenetic tree is based on core single nucleotide polymorphisms (SNPs), and was estimated using the Nullarbor pipeline (same sequence data as in Figure 2). British pig isolates are represented by blue circles at branch tips, while the vaccine strain is represented by a red star. SpaA group (1 or 2) is shown by the yellow/orange color strip. Serotype (1a, 1b, 2, 5) as determined *in silico* is shown by the purple/green color strip. Those where phenotypic testing was not performed or where *in silico* test results differed are not shown. Variants of 8 different immunogenic surface proteins are shown by the black/gray color strips for all British pig isolates. Those shown in black are those which cluster most closely with the variant found in the vaccine strain; isolates in dark gray represent isolates with surface protein variants in outlier groups (Supplementary Table S1, Supplementary File S1).

Among the 45 newly sequenced *E. rhusiopathiae* isolates from British pigs and the vaccine strain, a minimum mean sequencing depth of 26X was achieved, with a mean of 82X. The majority of the newly sequenced strains from British pigs fell within Clade 2 (32/45 = 71.1%), as did the vaccine strain (Figure 2). Five isolates (11.1%) were in Clade 3, while the remaining 17.8% (8/45) were within the intermediate clade. None of the isolates belonged to Clade 1. The average number of core SNP differences separating the pig isolates from one-another was 435 (median = 377, range 0–767). This was comparable to the number of core SNPs separating the vaccine strain from the field strains (median = 413, range 328–655). The core SNP profile of the vaccine strain was 100% identical across the two sequencing rounds. No clear geographic clustering was evident based on submitting veterinary center. Similarly, no obvious temporal clustering was apparent, indicating that rather than strain turnover, multiple divergent strains have remained in circulation throughout the study period (Figure 2). The genetic distance (based on core SNPs) of the British pig isolates from the vaccine strain did not change appreciably over time, thus providing no evidence for potential vaccine-induced selection (Supplementary File S1, Supplementary Figure S2). The phenotypically determined serotypes for the British pig isolates and vaccine were confirmed *in silico* in 40 of 46 isolates, with six discrepancies (Supplementary Table S1). However, upon re-testing, the phenotypic serotype of these isolates matched that predicted by *in silico* testing. Among the global collection, the Fujisawa reference strain was confirmed as serotype 1a *in silico*, while 19 isolates were designated as serotype 1b, 27 as serotype 2, 17 as serotype 5, and 12 had no BLAST hits for any of the primer pairs, suggestive of belonging to a serotype other than 1a, 1b, 2 or 5. This corroborated with the previously determined serotype for eight isolates, including one serotype 9 for which none of these primer pairs matched. Two mismatches between phenotypic and *in silico* serotype were found: 1. Isolate HC-585, previously classified as serotype 1a, did not have any primer pair matches; 2. Isolate Mew22, a serotype “N” (i.e. does not react with any of the panel of antisera), was classified as a serotype 1b. The reverse primer sequence for serotype 5 was found to consistently have one SNP difference with all serotype 5 isolates. The distribution of different serotypes within the phylogeny is shown in Figure 3, where there was limited correlation with Clade.

### Occurrence and Variability of Spa Proteins

The *spaA* gene was present in all the newly sequenced isolates, while *spaB* and *spaC* genes were not detected. In the translated SpaA protein sequences, substantial diversity was found within the immunoprotective or “hypervariable” domain, which spans amino acids 30–413 (Figure 1). All SpaA amino acid sequences (*n* = 343) were initially classified into the five SpaA groups previously described (Janßen et al., 2015) (Supplementary Table S3), which are based upon amino acid variants in this domain. Beyond the previously reported positions, three additional variable amino acid sites were present in at least five isolates, and thus considered discriminatory. These were variants at position 97 (N/I), position 109 (N/H), and position 139 (Q/K). There were also several amino acid variants (*n* = 53) present in fewer than five isolates (Supplementary Table S4). One isolate (20767, from a wild bird) had a highly divergent SpaA protein, with 19 unique amino acids. If these sites are not considered, there were 34 variable sites, four of which had two different amino acid variants (3 alleles).

**FIGURE 4:**
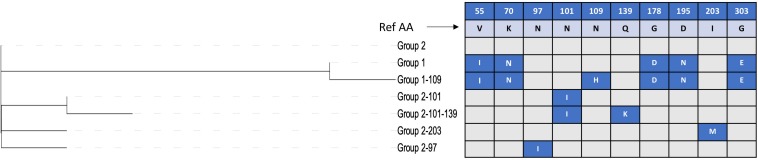
Discriminatory amino acid positions within the immunoprotective domain of the SpaA protein of *Erysipelothrix rhusiopathiae.* Amino acid (AA) variant positions with respect to the Fujisawa reference sequence are listed in the top row, with the amino acid found in the reference sequence shown in the second row. The phylogenetic tree shown to the left shows the relatedness among the SpaA sequence variants. Variants in each group are shown in blue boxes, with the AA change indicated.

**FIGURE 5:**
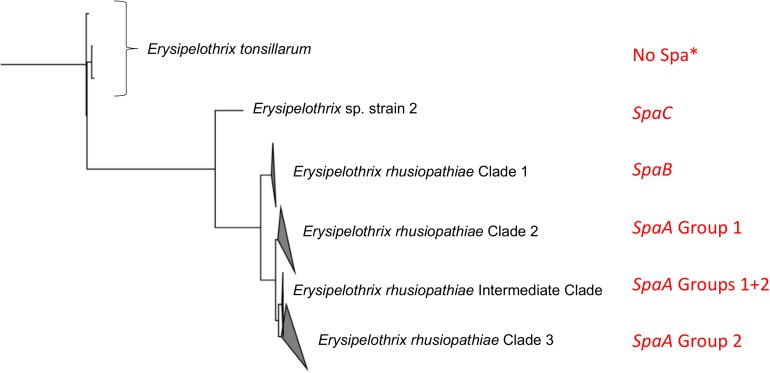
Relationship of Spa type and SpaA group to the population structure of *Erysipelothrix* spp. This figure is adapted from Forde T. et al., 2016. The tree is rooted to other genera of the family Erysipelotrichaceae. Spa type and SpaA group are indicated in red. SpaA group is based on discriminatory amino acid variants (Figure 4). *^∗^spaA and spaB have been reported only once each in Erysipelothrix tonsillarum* (Pomaranski et al., 2017).

The phylogenetic tree of the different SpaA protein variants suggests that there are two main groups (Figure 4), and we found that these groups are correlated with the overall population structure of *E. rhusiopathiae* (Figures 3, 5). We therefore propose a simplified nomenclature for SpaA groups, where Group 1 has amino acid variants at positions 55 (V/I), 70 (K/N), 178 (G/D), 195 (D/N) and 303 (G/E) in relation with the Fujisawa reference, consistent with the previous scheme proposed by Janßen et al. (2015). All other previously described groups are minor variants of what we now refer to as Group 2. These variants appear to arise multiple times throughout the phylogeny (Supplementary File S1, Supplementary Figure S3); this is suggestive of either parallel evolution (homoplasy) or recombination. A detailed description of how this SpaA classification relates to previously described groups is found in Supplementary Table S3 and Supplementary File S3.

All isolates within the SpaA group 1 belonged to Clade 2 or the intermediate clade. All Clade 2 isolates possessed SpaA group 1 except for a single British pig isolate (swine100, Figure 3). Since the majority of the British pig isolates belonged to Clade 2, SpaA group 1 was the variant found in the majority of these strains (82%; Supplementary Table S1). The vaccine strain was a variant of Group 1, with an additional amino acid difference at position 109 (N/H). This variant was also found in seven other sequences from GenBank; however, none of the British pig isolates investigated in this study shared this difference.

### Predicted Structure of SpaA

We aimed to assess the potential functional implications of variant residues detected within SpaA. However, given that the structure of SpaA is unknown and its function poorly understood, its structure was predicted as an exploratory exercise. A full-length structural model was produced using I-TASSER, a program that predicts structure using a combination of threading to existing protein structures and *ab initio* modeling. The majority of the highest ranked structural templates for the SpaA protein detected by I-TASSER were surface-located choline-binding proteins of *Streptococcus pneumoniae*, either phosphorylcholine esterase (Pce) also known as choline-binding protein E (CbpE) (PDB ID: 2BIB; Hermoso et al., 2005) or choline-binding protein F (CbpF) (PDB IDs: 2V04, 2V05; Molina et al., 2009). Other Firmicutes templates from *Clostridium difficile* and *Leuconostoc mesenteroides* were also identified. The highest-ranked structural model predicted using I-TASSER had a C-score of −1.30 indicating a good degree of confidence in the predicted structure (Roy et al., 2010).

Comparison with solved PDB structures showed that the structural model of *E. rhusiopathiae* SpaA most closely resembled the Pce protein of *S. pneumoniae* (Hermoso et al., 2005) (TM-score = 0.82, RMSD = 1.50 Å for 524 aligned Cα atoms, 17.6% amino acid sequence identity in structurally aligned region). The *S. pneumoniae* Pce comprises an N-terminal catalytic module (300 residues) and a C-terminal tail (228 residues) responsible for choline-binding (Hermoso et al., 2005). The hypervariable domain of the SpaA structural model (residues 30–413) aligned to the Pce catalytic module (TM-score = 0.75, RMSD = 1.88 Å for 303 aligned Cα atoms), while the C-terminal domain (residues 448–626) aligned to the Pce choline-binding domain (TM-score = 0.98, RMSD = 0.37 Å for 177 aligned Cα atoms).

**FIGURE 6:**
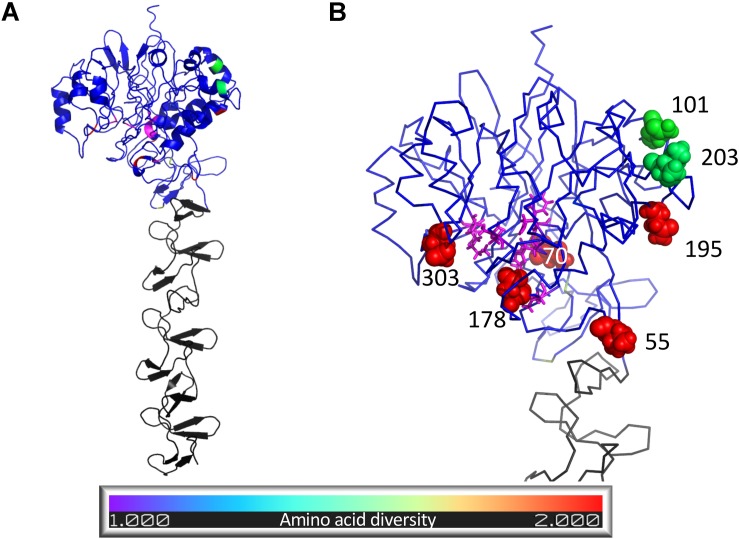
Structural model of *Erysipelothrix rhusiopathiae* SpaA protein predicted using I-TASSER. Residues structurally aligned to the active site of the *S. pneumoniae* Pce protein are colored magenta while the remaining residues of the N-terminal domain (residues 1–447) are colored following the legend according to amino acid diversity (Inverse Simpson index) calculated from 343 SpaA sequences sourced from newly sequenced isolates and public sequence databases. Residues of the C-terminal domain (residues 448–626) are colored gray. **(A)** Cartoon representation of whole structural model showing secondary structure. **(B)** Close up ribbon representation of N-terminal domain with spheres highlighting the nine residues with highest amino acid diversity (Inverse Simpson index > 1.3) and with residues structurally aligned to the active site of the *S. pneumoniae* Pce protein (magenta) shown in stick format. Figure created using Pymol (Schrödinger).

While the function of the N-terminal domain is unknown, we hypothesized that it might be similar to that of its closest homolog, which we found to be the Pce protein of *S. pneumoniae*. As such, we examined the proximity of variable SpaA positions to the residues of the structural model homologous to those comprising the Pce catalytic site. Residues of the *S. pneumoniae* Pce protein catalytic domain active site were mapped to the SpaA structural model using TM-align so that the proximity of variable SpaA sites to the potential active site could be calculated. The structural model of the *E. rhusiopathiae* SpaA protein is shown in Figure 6, colored according to amino acid diversity calculated across the alignment of available sequences. Of the residues with the highest amino acid diversity (55, 70, 101, 178, 195, 203, 303, 426, and 435, Inverse Simpson index > 1.3), positions 70, 178 and 303 were closest to residues of the potential active site with distances of under 10 Å (for comparison, the distance between covalently bound residues was 3.5–4 Å). For position 109, the position at which most *E. rhusiopathiae* sequences from British pigs differ relative to the vaccine (Asn – His), the lowest distance to a residue aligned to the Pce protein catalytic domain active site was 13.3 Å which is beyond the range expected to be structurally impacted by a mutation between His and Asn at that position.

The extent to which SpaA is recognized by antibodies of the humoral immune system is not known. To investigate the potential for variant residues to influence antibody recognition, the degree to which their location within the structural model showed epitope characteristics was assessed. Thus, to evaluate the potential of the variant residues influencing recognition by the humoral immune system, the structural model was used to generate epitope scores that reflect the likelihood of each residue belonging to a B-cell epitope. Epitope scores, which reflect the likelihood of each residue belonging to a B-cell epitope, were calculated for each residue within the SpaA structural model based on surface exposure, amino acid identity and side chain orientation (Sweredoski and Baldi, 2008). Of the nine high-diversity positions, 55 and 435 were both placed among the highest predicted epitope scores, at the 94th and 93rd percentile, respectively, but there was no consistent relationship between diversity and epitope scores. The epitope score associated with position 109 on the SpaA was at only the 8th percentile calculated across all positions, suggesting the position is unlikely to be important for antibody recognition. For all positions, amino acid diversity, predicted secondary structure, epitope score, and aligned *S. pneumoniae* Pce protein active site residues are detailed in Supplementary Table S6.

### Occurrence and Variability of Other Surface Proteins

Of the 12 other *E. rhusiopathiae* surface proteins examined, 11 were present in all isolates from British pigs and the vaccine (i.e. were core genes). However, the degree to which amino acid sequences were conserved varied greatly among proteins (Table 2; Supplementary Table S5; Supplementary File S2). Of the surface proteins examined, GAPDH was the most conserved, with only a single variant across the 334 amino acid protein, found in a single isolate (swine74). Atsp and Da were also highly conserved, with 6 and 13 variant positions, respectively. It is noteworthy that three of the six variant positions in Atsp were unique to the vaccine strain. Of the 52 variant positions identified in Bga, three of these were unique to the vaccine strain. One isolate (swine98) also had a mutation conferring a premature stop codon for this protein. A slightly higher proportion of sites varied in Neu (3.4%), Bml (3.8%) and rspA (4.8%); the latter had four sites unique to the vaccine. CbpA showed much higher diversity, with variants at 162 of 1773 amino acid positions (9.1%), but none of which were unique to the vaccine.

**TABLE 2:** Amino acid (AA) sequence diversity in surface proteins related to immunogenicity of *Erysipelothrix rhusiopathiae.*

	**Protein**				**BLAST hit results where coding**
	**length**	# Variant AAs*	**# variant AAs**	**Insertions/deletions and**	** sequence was spread across**
**Surface protein**	**(AAs)**	**(% of total AAs)**	**unique to vaccine**	**premature stop codons**	**multiple contigs^+^**
*rspA*	1992	96 (4.8%)	4		swine40 (3 contigs) swine94 (3 contigs)
*cbpB*	606	27 (4.5%)	1	Deletion 20AAs swine78 (1 repeat unit)	
GAPDH (*gapA*)	334	1 (0.3%)	0		
Dipeptidyl aminopeptidase (*Da, dap2*)	657	13 (2%)	0		
*CbpA*	1773	162 (9.1%)	0		
*Plp*	1519–1531	91 (6%)	0	Insertion of either 4 AAs (16 strains) or 12 AAs (2 strains)	swine40 (2 contigs)
*Atsp*	481	6 (1.2%)	3		swine50 (2 contigs)
Neuraminidase (*Neu, nanH*)	1204	41 (3.4%)	1		
Beta-galactosidase (*Bga*)	2091	52 (2.6%)	3	swine98 stop codon at AA Pos 658	swine40 (3 contigs) swine50 (2 contigs) swine94 (3 contigs)
*Bml*	364	14 (3.8%)	0		swine40 (2 contigs)
*CwpA*	2134	N/A	N/A		Full length BLAST hits in 13 isolates (5322 bp) Missing stop codon in 6 isolates Remaining 27 isolates with BLAST hits ranging from 758 – 3116 bp (median = 2610)
*CwpB*	1009–1020	276 (27%)	1	Insertion 7AAs swine18 Deletion 4AAs swine103	swine40 (2 contigs)

Isolates were examined from British pigs (*n* = 45) and a commercially available vaccine strain in comparison with the Fujisawa reference strain. * Comparisons are made in relationship with reference strain Fujisawa (NC_015601). ^+^Complete coding sequences were found in all isolates (based on BLASTn searches of the *de novo* assembled nucleotide sequences) unless otherwise stated. bp = base pairs.

The only proteins with any insertions or deletions identified among the British pig isolates were CbpB, Plp and CwpB. In CbpB, one isolate (swine78) had a deletion of 20 amino acids, corresponding to the loss of a repeat unit. In Plp, an insertion of either 4 amino acids (16 strains) or 12 amino acids (two strains) was present. In CwpB, a single isolate (swine103) had a deletion of 4 amino acids (positions 971–974), while another isolate (swine18) had an insertion of 7. This protein also had the highest proportion of variant positions (27%), although the majority of the variant sites (83%) were due to two isolates whose sequences differed greatly from the others (swine18 and swine101; Supplementary Table S5 and Supplementary File S2). Finally, one surface protein examined was variably present among the isolates. Complete nucleotide sequences for CwpA (5322 bp) were present in only 13 isolates, as well as in an additional 6 isolates that were missing a stop codon. The remaining 27 isolates had BLAST hits for this sequence ranging from 758 – 3116 bp (median = 2610); the vaccine strain had a hit length of 3116 bp. Some of the BLAST hits for the surface proteins investigated were only partial sequences spread across multiple contigs (i.e. had not been completely assembled) (Table 2). The affected isolates had lower quality assemblies overall (swine94, 50, and 40), as shown by the lowest N50 values among the newly sequenced isolates (Supplementary Table S1).

The phylogenetic trees generated for the different surface proteins (Supplementary File S2) did not show concordance with the core SNP phylogeny. This is illustrated in Figure 3, where for each surface protein, the isolates that cluster most closely with the protein sequence of the vaccine strain are shown. There were no major changes to the distribution of the different surface protein variants over the timeframe of the study period (Supplementary File S1, Supplementary Figure S4).

## Discussion

### Diversity of Circulating *E. rhusiopathiae* Strains in Pigs in Great Britain and Their Relation to a Commonly Used Vaccine Strain

While the global genomic diversity of *E. rhusiopathiae* has been characterized, how much of this genomic and functional diversity is maintained at smaller scales was unclear. This study provides a valuable contribution to our understanding of *E. rhusiopathiae* genomics, and in particular provides novel data on the diversity of surface antigens. Moreover, fully sequencing a commercial vaccine strain of *E. rhusiopathiae* has provided valuable insights into its relationship to circulating field strains.

Throughout the 27-year time period from which samples were collected for this study (1987 – 2014), phylogenetically diverse strains of *E. rhusiopathiae* were isolated from British pigs (Figure 2). Similarly high diversity was previously observed in poultry isolates from Germany based on multi-locus sequence typing (Janßen et al., 2015), while the diversity of *E. rhusiopathiae* circulating in pigs in Asia has been shown to be restricted to strains within the intermediate clade (Ogawa et al., 2017). The lower diversity of *E. rhusiopathiae* in Asian countries could be due a more recent introduction of the pathogen, and/or differences in animal trade, pig breeding, herd management or biosecurity. The majority of the British pig isolates belong to Clade 2, which differs from what was previously found in North America, where all pig and poultry isolates investigated (*n* = 14) belonged to Clade 3 (Forde T. et al., 2016), suggesting different *E. rhusiopathiae* clades have become dominant on these continents. The vaccine strain also belonged to Clade 2. We found no evidence that *E. rhusiopathiae* strains in circulation in Great Britain have diverged from the vaccine strain over the study period. It would have been interesting to investigate the strains associated with other commercially available vaccines, however, we were unable to detect and extract *E. rhusiopathiae* DNA from the second vaccine tested. Whether there is a component of the vaccine adjuvant (e.g. aluminum hydroxide) that inhibited detection of DNA by Qubit and PCR is unknown. None of the isolates we sequenced from British pigs in this study belonged to *E. rhusiopathiae* Clade 1, emphasizing the rarity of strains from this clade causing disease in the major production species worldwide; in a global collection of isolates previously sequenced (Forde T. et al., 2016), only 8% (7/86) belonged to Clade 1, and none of these were from pigs or poultry.

### Spa Type and Correlation With Population Structure and Clinical Disease

No *spaB* or *spaC* genes were found among the *E. rhusiopathiae* isolates from British pigs. Given the lack of Clade 1 isolates, this finding was expected and is consistent with previous observations that Clade 1 isolates carry the *spaB* gene, whereas Clade 2, 3 and intermediate isolates carry the *spaA* gene (Figure 5; Forde T. et al., 2016). To date, *spaC* has only been associated with *Erysipelothrix* sp. strain 2, and not *E. rhusiopathiae*. The predominance of the *spaA* gene in isolates associated with clinical erysipelas has been supported in several studies (Eamens et al., 2006; To et al., 2012; Janßen et al., 2015). It therefore seems that *spaB* and *spaC* (i.e. isolates belonging to *E. rhusiopathiae* Clade 1 and *E*. sp. Strain 2) are less relevant when considering vaccine design for production species. However, given that *E. rhusiopathiae* Clade 1 is more commonly isolated from marine mammals in captivity (Opriessnig et al., 2013; Forde T. et al., 2016), and *Erysipelothrix* spp. carrying the *spaC* gene has recently been associated with disease in fish (Pomaranski et al., 2017), protecting against *Erysipelothrix* spp. carrying these Spa types could be of greater relevance for aquatic species. It was previously suggested that Spa proteins are likely important virulence factors associated with the pathogenic potential of *E. rhusiopathiae* in comparison to the less pathogenic *E. tonsillarum* (To and Nagai, 2007), and until recently, no Spa-related genes or proteins had been found in *E. tonsillarum* (To and Nagai, 2007; Shen et al., 2010). To our knowledge, the occurrence of *spaA* and *spaB* genes in *E. tonsillarum* has only been documented in one paper, among isolates from ornamental fish (Pomaranski et al., 2017). However, given that the species was defined based only on sequencing of the *gyrB* gene, it is conceivable that this isolate may have been misclassified.

### SpaA Diversity, Correlation With Population Structure, and Relevance for Vaccination and Pathogenicity

This study represents the largest examination of the diversity of SpaA protein sequences in *E. rhusiopathiae* conducted to date, based on a collection of publicly available sequences and *de novo* whole genome sequence assemblies (*n* = 343). With this extensive collection of sequences, we were able to identify additional amino acid variants that could further discriminate SpaA protein sequences in comparison to previous typing schemes (Uchiyama et al., 2014; Janßen et al., 2015). We were also able to assess for correlation of these groups with population structure (Figure 5). SpaA group 1 was only present in Clade 2 and the intermediate clade, but was absent from the Clade 3 isolates examined (Figure 3). Janßen et al. (2015) found a strong correlation between SpaA group 1 and their ST complex 9. It is very likely that ST-9 corresponds with Clade 2, since we found that all Clade 2 isolates had this SpaA variant with one exception. We propose amalgamating all other previously described SpaA groups into a single group, forthwith referred to as SpaA group 2, since variants within this group are limited to only one or two amino acid differences. Moreover, these variants appear to be unconstrained by the core phylogeny (Supplementary File S1). Such incongruent patterns could arise either through parallel evolution (homoplasy), or, more likely, through horizontal gene transfer, given the apparent propensity for recombination observed in this species.

Whether the observed differences within the SpaA protein could be responsible for differential immune protection – or for differences in virulence – remains an important area for future study. For instance, the vaccine strain tested in this study belonged to Group 1 SpaA along with the majority of British pig isolates, but had an amino acid change from N→H at position 109. Whether this could result in differential immune response is unknown; however, based on the predicted structure of SpaA, we found that this position is unlikely to represent an epitope.

Isolates with methionine at position 203 (i.e. Janßen Group 5, Uchiyama Group 1) were rare among the isolates examined in this study. This variant was present in one British pig isolate (Supplementary Tables S1, S3) and in three WGS available on GenBank (GXBY-1, WH13013 and ML101), but in none of the global collection of isolates (*n* = 75). It was previously found in a handful of pig isolates from Germany (*n* = 4) (Janßen et al., 2015). Otherwise, this variant has been mostly limited to strains from China (Supplementary Table S2) and Japan, where it is widespread and has been isolated from pigs with acute, subacute and chronic infections (To et al., 2012; Uchiyama et al., 2017) and where it appears to be increasing in prevalence (Ogawa et al., 2017). There is, however, not yet any evidence of this variant being associated with an increase in virulence (Zou et al., 2015). One particular variant that would be worthy of further investigation is that of alanine at position 195 (previously classified as Group 3 by Uchiyama et al., 2014). This is the variant carried by the pathogenic reference strain SY1027 from China (Kwok et al., 2014), and was the variant carried by the *E. rhusiopathiae* strain associated with large-scale muskox die-offs in northern Canada (Kutz et al., 2015; Forde T.L. et al., 2016). This variant has also been reported among pigs in Japan, although is apparently being replaced by newer lineages (Ogawa et al., 2017). Whether this amino acid change confers increased virulence would be valuable to explore.

### Predicted SpaA Protein Structure

We found that the amino acid sequence of the *E. rhusiopathiae* SpaA protein predicts a protein structure most closely resembling that of choline-binding proteins of *Streptococcus pneumoniae.* Indeed, the homology in the C-terminal region of these proteins had previously been described (Borrathybay et al., 2015). This region – containing tandem repeats including the GW motif – is a motif conserved across Gram-positive bacteria that allows proteins to bind to choline residues of techoic acid on the cell surface (Jedrzejas, 2001). It is believed that this motif is what allows SpaA to bind to phosphorylcholine of the of *E. rhusiopathiae* capsule (Harada et al., 2014), facilitating binding to host endothelial cells. To our knowledge, no studies have previously examined the structure of the N-terminal region of SpaA, which is the portion shown to be involved in its protective immunogenicity. Among proteins for which the structure has been determined, we found the N-terminal portion of the *S. pneumoniae* Pce protein was the closest match, although with lower homology in comparison with the C-terminal portion. *S. pneumoniae* Pce modifies the distribution of phosphoryl choline on the bacterial surface, impairing the ability of the host immune system to efficiently bind, providing a mechanism for immune escape (Hermoso et al., 2005). Given the predicted structural similarity, it is possible that SpaA has a similar function, though this hypothesis would need to be further explored through functional assays.

Working with the hypothesis that SpaA may have a similar function to *S. pneumoniae* Pce, we explored whether SpaA positions with high amino diversity, including those differing between groups 1 and 2, were located near to a possible active site. The N-terminal module of the Pce protein contains a binuclear Zn2 + catalytic center (Hermoso et al., 2005). Within this active center, particular residues have been shown to be involved in substrate binding and catalysis, facilitating teichoic acid hydrolysis which releases phosphoryl choline moieties reducing recognition by the immune system. In SpaA, the variant amino acid residues at positions 70, 178 and 303 were those closest to potential active site, as based on homology with the *S. pneumoniae* Pce protein. Since these are all variants that distinguish between SpaA groups 1 and 2, it is possible that these variants confer functional differences between these groups.

Alternatively, we considered that variable positions may have played a role in recognition by the humoral immune system. To do so, we assessed whether diverse amino acid sites tended to occupy locations within the structural model that exhibited signatures of epitope regions. With the notable exception of position 55, a variant that differs between groups 1 and 2, and position 435 that possessed very high predicted epitope scores, the diverse positions did not show a general tendency toward high epitope scores, suggesting pressure from the humoral immunity may not be a major driver of the observed genetic diversity. The role of SpaA in immune recognition has not been characterized in great detail. *In vitro* experiments and challenge studies to assess cross-protection, wherein isolates from different SpaA groups are included, would be valuable.

### Variability in Immunogenic Surface Proteins

Our study provides novel information about the sequence diversity of different immunogenic surface proteins of *E. rhusiopathiae*. The degree of variability differed substantially among the 12 proteins we investigated, ranging from proteins that were nearly completely conserved across all 45 isolates and the vaccine strain (GAPDH) to 27% of the amino acids showing variation (CwpB), as well as some proteins with small insertions and deletions (Plp and CwpB). Coding sequences for these proteins were found to be core genes, with the exception of CwpA which was variably present. Based on the phylogenetic trees estimated for each protein (Supplementary File S1), variants of the different proteins did not correlate with the phylogenetic relationship among isolates (Figure 3), suggesting that horizontal transfer of genes encoding for antigens is commonplace for *E. rhusiopathiae*.

We investigated whether there was any evidence of antigenic divergence (Bart et al., 2014) between field strains of *E. rhusiopathiae* and a common vaccine strain, as this could potentially be of concern for vaccine efficacy. However, we did not observe any such temporal shift in surface protein variants for any of the antigens we examined (Supplementary File S1). The vaccine strain had unique amino acids in 5 of the 12 surface proteins examined – rspA, Bga, Atsp, Neu and CwpB (i.e. that were not present among the 45 pig isolates). Of particular note is in Atsp, where this included three of only six variants across the protein and among all isolates. The impact of these differences – if any – would be valuable to explore. The most conserved surface protein – GAPDH – has been confirmed to be expressed at the cell surface (Shi et al., 2013), and like SpaA, RspA and RspB, has a role in endothelial adhesion (Zhu et al., 2017c). It was recently found to provide good protection in both murine and porcine challenge models (Zhu et al., 2018). These authors suggested that given its proven role as a protective antigen, in addition to being a housekeeping gene and thus highly conserved – as confirmed by this study – GAPDH could make a good candidate for a subunit vaccine. Indeed, subunit vaccines based on surface-exposed proteins have been proposed as a potential alternative to traditional live or bacterin-based vaccines (Hu et al., 2017).

### Serotype as an Epidemiological and Immunological Marker

*E. rhusiopathiae* isolates have historically been described by their serotype, which is based on testing for agglutination of an isolate against a panel of serotype-specific antisera using a double agar-gel diffusion method (Kucsera, 1973). It was recently determined that a chromosomal region encoding a putative pathway for polysaccharide biosynthesis is responsible for defining the antigenicity of the major serotypes 1a, 1b, 2 and 5, and that these can be distinguished based on their genetic composition at this locus (Ogawa et al., 2018; Shiraiwa et al., 2018). However, this present study lends further support to the fact that serotype is inappropriate for assessing the genetic relatedness among isolates at larger scales (e.g. at a national level). It has even been reported that the same serotypes can be found across multiple *Erysipelothrix* species (Takahashi et al., 2008; McNeil et al., 2017). Given the lack of phylogenetic informativeness of serotype, it may not be the most relevant feature by which to primarily classify isolates. Rather, a hierarchical approach should be taken (i.e. Figure 5), wherein *Erysipelothrix* spp. isolates are described by species, clade and/or Spa type, followed by SpaA group and serotype. The value of serotype as an epidemiological marker at smaller scales (e.g. within or between farm spread) would be valuable to assess.

Whether serotype is a relevant trait for predicting cross-protection among strains remains unknown and warrants further study. It has been suggested that vaccines based on serotype 2 strains confer protection against serotype 1 and 2 strains in pigs, as well as other serotypes to a variable degree (Wood et al., 1981; Takahashi et al., 1984; Sawada and Takahashi, 1987; Kitajima et al., 1998). Since serotypes 1 and 2 are those most commonly associated with clinical disease globally (Wood and Harrington, 1978; Cross and Claxton, 1979; Takahashi et al., 1996; Opriessnig et al., 2004; Coutinho et al., 2011; McNeil et al., 2017), these challenge studies suggest that currently available vaccines should confer protective immunity against a variety of strains. However, since these pig challenge studies have only included one representative strain per serotype, the potential for confounding is very high (i.e. the possibility that these findings are based on immunogenic features other than serotype), making it difficult to assess the likelihood or relevance of serotype-associated cross protection.

The newly reported PCR scheme (Shiraiwa et al., 2018) for distinguishing among serotypes 1a, 1b, 2 and 5 was successfully applied *in silico* to determine the serotype of sequenced isolates for which serotype was not previously known. Initial discrepancies between phenotypic and *in silico* results for some British pig isolates were resolved upon re-running the agglutination tests on fresh subcultures, suggesting that the *in silico* method may produce more accurate and reproducible results than standard methods. However, as the authors of this PCR scheme previously noted and as we found for global isolate MEW22, one limitation is that certain serovars that would be phenotypically untypeable (“N”) using the double agar-gel diffusion method may be assigned to a serotype. This likely represents the original serotype that no longer yields serotype-specific antigen due to only minor genetic differences that result in changes in antigen-antibody reactions. Further studies will be valuable to expand our understanding of the genetic basis for other serotypes and the molecular tools available for distinguishing them. We found that the reverse primer sequence for serotype 5 had one nucleotide discrepancy with all isolates of this serotype in the global collection (*n* = 17); this sequence should therefore be updated to 5′-GAAATAATGCC**A**ATAGATGGAGCACC-3′.

### Vaccine Cross Protection

Vaccination success is multifactorial, and includes factors related to delivery (e.g. maintenance of cold chain), host-related factors (e.g. age, health status, genetic factors), and factors related to the pathogen. This study highlights important knowledge gaps associated with pathogen-related factors for *E. rhusiopathiae* vaccination, namely what are the determinants of protective immunity, and what features confer cross-protection. Protection induced by *E. rhusiopathiae* vaccination is via both cell-mediated and humoral immunity (Shimoji, 2000; Pomorska-Mól et al., 2012). Protective immunity is more difficult to elucidate, as it can only be assessed by challenge studies. It is likely stimulated via a complex array of antigens, including proteins and polysaccharides. Ultimately, it would be valuable for producers and clinicians to know what vaccine(s) would be suitable to provide protection against the specific strains found on a farm, but information to make such a judgment is currently lacking. The surface proteins explored in this study have all been shown to be immunogenic, but whether differences in their structure or their presence or absence can impact a strain’s ability to confer a protective immune response against different strains remains unknown. In most challenge studies that have been conducted in both mice and pigs, the majority of *E. rhusiopathiae* vaccines have provided a protective immune response against a range of different strains. The key question for the field may therefore be ‘What genetic and/or antigenic differences between strains would result in a *failure* to provide cross protection?’. There is evidence that the most common vaccine strains are not consistently protective against strains assigned to serotypes 9 or 10 (Wood, 1979; Wood et al., 1981; Takahashi et al., 1984), however, these strains are likely sufficiently rare in field conditions to make this a minor concern (Haesebrouck et al., 2004). It has been well recognized that gram-positive bacterial cell wall antigens, including lipoteichoic acid, have immunomodulating properties (Shimoji et al., 2019). It is possible that serotype differences in *E. rhusiopathiae* result in different immunostimulatory potencies (i.e. have a role as adjuvants), but this requires further studies for confirmation. Whether phylogenetic distance (e.g. differences in clade) impacts the ability of a vaccine to confer protective immunity is an important area of study that has yet to be explored. For instance, it is not known whether the vaccine strain (Clade 2) would be protective against more divergent isolates from Clade 3. Similarly, the relevance of surface protein variants – including SpaA groups – for immune cross-protection is unknown. This could initially be explored using serum from immunized pigs, and testing for antibody responses against different strains through ELISA. A further complication is the possibility of mixed infection, which we previously showed to be quite common for *E. rhusiopathiae* infection, at least in wild ungulates (Forde T.L. et al., 2016). If a host were infected with multiple strains, particular variants might gain hold if they are undetected by the immune system. The inclusion of multiple strains in a single vaccine could potentially overcome some of these issues.

## Conclusion

In this study, we show that diverse *E. rhusiopathiae* strains have been circulating in pigs in Great Britain over the past few decades, and that the average genetic distance of these field strains from the vaccine strain has remained relatively stable over time. This study provides further support that SpaA is the most relevant Spa type associated with clinical erysipelas in production species (i.e. pigs and poultry); suggestions to update the nomenclature related to SpaA groups based on amino acid variants are provided. We provide novel data on the degree of conservation of various immunogenic surface proteins, and demonstrate that horizontal gene transfer has likely contributed to the observed diversity. Research into which variables are relevant for conferring cross-protection will be critical for understanding the relevance of this diversity for vaccine development. As the agricultural industry is working to reduce the use of antibiotics due to increasing concerns related to antimicrobial resistance, the availability and selection of effective vaccines is increasingly important. Variants identified in this study could serve as a basis for guiding the selection of strains to be included in future *in vitro* and *in vivo* challenge studies.

### Commercial Products Used

The sequenced vaccine was Porcilis Ery^®^, MSD Animal Health, which contains serotype 2 strain M2. This strain, either as a monovalent vaccine or in combination with vaccines against parvovirus ± *Leptospira* spp., reportedly accounts for more than half of the total market in United Kingdom vaccines against *E. rhusiopathiae* (R. Warin, Hipra, personal communication). The second vaccine from which DNA was not successfully extracted was Eryseng^®^, Hipra.

## Data Availability Statement

The sequence data generated for this study can be found in the European Nucleotide Archive under accession number PRJEB34953.

## Author Contributions

TF and TO conceived, designed, and coordinated the study. JT and SW provided sample collections and associated metadata. TF performed laboratory work and drafted the manuscript. TF, NK, WH, and RB performed and/or advised on data analysis. All authors contributed to editing the manuscript and approved the final version.

## Conflict of Interest

The authors declare that the research was conducted in the absence of any commercial or financial relationships that could be construed as a potential conflict of interest.

## Footnotes

1 https://github.com/tseemann/nullarbor

2 https://pymol.org

## References

[B1] Bankevich A. Nurk S. Antipov D. Gurevich A. A. Dvorkin M. Kulikov A. S. (2012). SPAdes: a new genome assembly algorithm and its applications to single-cell sequencing. *J. Comput. Biol.* 19 455–477. 10.1089/cmb.2012.0021 22506599 PMC3342519

[B2] Bart M. J. Harris S. R. Advani A. Arakawa Y. Bottero D. Bouchez V. (2014). Global population structure and evolution of *Bordetella pertussis* and their relationship with vaccination. *mBio* 5:e01074. 10.1128/mBio.01074-14 24757216 PMC3994516

[B3] Bolger A. M. Lohse M. Usadel B. (2014). Trimmomatic: a flexible trimmer for Illumina sequence data. *Bioinformatics* 30 2114–2120. 10.1093/bioinformatics/btu170 24695404 PMC4103590

[B4] Borrathybay E. Gong F.-J. Zhang L. Nazierbieke W. (2015). Role of surface protective antigen A in the pathogenesis of *Erysipelothrix rhusiopathiae* strain C43065. *J. Microbiol. Biotechnol.* 25 206–216. 10.4014/jmb.1407.07058 25223326

[B5] Connor T. R. Loman N. J. Thompson S. Smith A. Southgate J. Poplawski R. (2016). CLIMB (the Cloud Infrastructure for Microbial Bioinformatics): an online resource for the medical microbiology community. *BioRxiv* [preprint] 10.1101/064451 PMC5537631 28785418

[B6] Coutinho T. A. Imada Y. Barcellos D. E. S. N. Oliveira S. J. Moreno A. M. (2011). Phenotypic and molecular characterization of recent and archived *Erysipelothrix* spp. isolated from Brazilian swine. *Diagn. Microbiol. Infect. Dis.* 69 123–129. 10.1016/j.diagmicrobio.2010.09.012 21251554

[B7] Cross G. M. Claxton P. D. (1979). Serological classification of Australian strains of *Erysipelothrix rhusiopathiae* isolated from pigs, sheep, turkeys and man. *Aust. Vet. J.* 55 77–81. 10.1111/j.1751-0813.1979.tb15170.x 444165

[B8] Eamens G. J. Forbes W. A. Djordjevic S. P. (2006). Characterisation of *Erysipelothrix rhusiopathiae* isolates from pigs associated with vaccine breakdowns. *Vet. Microbiol.* 115 329–338. 10.1016/j.vetmic.2006.02.015 16621346

[B9] Edgar R. C. (2004). MUSCLE: multiple sequence alignment with high accuracy and high throughput. *Nucleic Acids Res.* 32 1792–1797. 10.1093/nar/gkh340 15034147 PMC390337

[B10] Eriksson H. Nyman A.-K. Fellström C. Wallgren P. (2013). Erysipelas in laying hens is associated with housing system. *Vet. Rec.* 173:18. 10.1136/vr.101388 23542656

[B11] Forde T. Biek R. Zadoks R. Workentine M. L. De Buck J. Kutz S. (2016). Genomic analysis of the multi-host pathogen *Erysipelothrix rhusiopathiae* reveals extensive recombination as well as the existence of three generalist clades with wide geographic distribution. *BMC Genomics* 17:461. 10.1186/s12864-016-2643-0 27301771 PMC4906694

[B12] Forde T. L. Orsel K. Zadoks R. N. Biek R. Adams L. G. Checkley S. L. (2016). Bacterial genomics reveal the complex epidemiology of an emerging pathogen in arctic and boreal ungulates. *Front. Microbiol.* 7:1759 10.3389/fmicb.2016.01759 PMC5097903 27872617

[B13] Gamberini M. Gómez R. M. Atzingen M. V. Martins E. A. L. Vasconcellos S. A. Romero E. C. (2005). Whole-genome analysis of *Leptospira interrogans* to identify potential vaccine candidates against leptospirosis. *FEMS Microbiol. Lett.* 244 305–313. 10.1016/j.femsle.2005.02.004 15766783

[B14] Gurevich A. Saveliev V. Vyahhi N. Tesler G. (2013). QUAST: quality assessment tool for genome assemblies. *Bioinformatics* 29 1072–1075. 10.1093/bioinformatics/btt086 23422339 PMC3624806

[B15] Haesebrouck F. Pasmans F. Chiers K. Maes D. Ducatelle R. Decostere A. (2004). Efficacy of vaccines against bacterial diseases in swine: what can we expect? *Vet. Microbiol.* 100 255–268. 10.1016/j.vetmic.2004.03.002 15145504

[B16] Harada T. Ogawa Y. Eguchi M. Shi F. Sato M. Uchida K. (2014). Phosphorylcholine and SpaA, a choline-binding protein, are involved in the adherence of *Erysipelothrix rhusiopathiae* to porcine endothelial cells, but this adherence is not mediated by the PAF receptor. *Vet. Microbiol.* 172 216–222. 10.1016/j.vetmic.2014.04.012 24856134

[B17] Hermoso J. A. Lagartera L. González A. Stelter M. García P. Martínez-ripoll M. (2005). Insights into pneumococcal pathogenesis from the crystal structure of the modular teichoic acid phosphorylcholine esterase Pce. 12 533–538. 10.1038/nsmb940 15895092

[B18] Hu Y.-F. Zhao D. Yu X.-L. Hu Y.-L. Li R.-C. Ge M. (2017). Identification of bacterial surface antigens by screening peptide phage libraries using whole bacteria cell-purified antisera. *Front Microbiol* 8:82 10.3389/fmicb.2017.00082 PMC5266700 28184219

[B19] Imada Y. Goji N. Ishikawa H. Kishima M. Sekizaki T. (1999). Truncated surface protective antigen (SpaA) of *Erysipelothrix rhusiopathiae* serotype 1a elicits protection against challenge with serotypes 1a and 2b in pigs. *Infect. Immun.* 67 4376–4382. 10.1128/iai.67.9.4376-4382.1999 10456877 PMC96755

[B20] Janßen T. Voss M. Kühl M. Semmler T. Philipp H.-C. Ewers C. (2015). A combinational approach of multilocus sequence typing and other molecular typing methods in unravelling the epidemiology of *Erysipelothrix rhusiopathiae* strains from poultry and mammals. *Vet. Res.* 46:84. 10.1186/s13567-015-0216-x 26198736 PMC4509749

[B21] Jedrzejas M. J. (2001). Pneumococcal virulence factors: structure and function. *Microbiol. Mol. Biol. Rev.* 65 187–207. 10.1128/MMBR.65.2.187-207.2001 11381099 PMC99024

[B22] Kearse M. Moir R. Wilson A. Stones-Havas S. Cheung M. Sturrock S. (2012). Geneious Basic: an integrated and extendable desktop software platform for the organization and analysis of sequence data. *Bioinformatics* 28 1647–1649. 10.1093/bioinformatics/bts199 22543367 PMC3371832

[B23] Kitajima T. Oishi E. Amimoto K. Ui S. Nakamura H. Okada N. (1998). Protective effect of NaOH-extracted *Erysipelothrix rhusiopathiae* vaccine in pigs. *J. Vet. Med. Sci.* 60 9–14. 10.1292/jvms.60.9 9492354

[B24] Kucsera G. (1973). Proposal for standardization of the designations used for serotypes of *Erysipelothrix rhusiopathiae* (Migula) Buchanan. *Int. J. Syst. Bacteriol.* 23 184–188. 10.1099/00207713-23-2-184

[B25] Kutz S. Bollinger T. Branigan M. Checkley S. Davison T. Dumond M. (2015). *Erysipelothrix rhusiopathiae* associated with recent widespread muskox mortalities in the Canadian arctic. *Can. Vet. J.* 56 560–563. 26028673 PMC4431149

[B26] Kwok A. H. Li Y. Jiang J. Jiang P. Leung F. C. (2014). Complete genome assembly and characterization of an outbreak strain of the causative agent of swine erysipelas – *Erysipelothrix rhusiopathiae* SY1027. *BMC Microbiol.* 14:176. 10.1186/1471-2180-14-176 24993343 PMC4105556

[B27] Makino S. Yamamoto K. Murakami S. Shirahata T. Uremura K. Sawada T. (1998). Properties of repeat domain found in a novel protective antigen. SpaA, of *Erysipelothrix rhusiopathiae*. *Microb. Pathog.* 25 101–109. 10.1006/mpat.1998.0216 9712689

[B28] McNeil M. Gerber P. F. Thomson J. Williamson S. Opriessnig T. (2017). Serotypes and Spa types of *Erysipelothrix rhusiopathiae* isolates from British pigs (1987 to 2015). *Vet. J.* 225 13–15. 10.1016/j.tvjl.2017.04.012 28720292

[B29] Molina R. González A. Stelter M. Inmaculada P. Kahn R. Morales M. (2009). Crystal structure of CbpF, a bifunctional choline-binding protein and autolysis regulator from *Streptococcus pneumoniae*. *EMBO Rep.* 10 246–251. 10.1038/embor.2008.245 19165143 PMC2658566

[B30] Ogawa Y. Ooka T. Shi F. Ogura Y. Nakayama K. Hayashi T. (2011). The genome of *Erysipelothrix rhusiopathiae*, the causative agent of swine erysipelas, reveals new insights into the evolution of firmicutes and the organism’s intracellular adaptations. *J. Bacteriol.* 193 2959–2971. 10.1128/JB.01500-10 21478354 PMC3133210

[B31] Ogawa Y. Shiraiwa K. Nishikawa S. Eguchi M. Shimoji Y. (2018). Identification of the chromosomal region essential for serovar-specific antigen and virulence of serovar 1 and 2 strains of *Erysipelothrix rhusiopathiae*. *Infect. Immun.* 86:e00324-18. 10.1128/IAI.00324-18 29891546 PMC6105884

[B32] Ogawa Y. Shiraiwa K. Ogura Y. Ooka T. Nishikawa S. Eguchi M. (2017). Clonal lineages of *Erysipelothrix rhusiopathiae* responsible for acute swine erysipelas in japan identified by using genome-wide single-nucleotide polymorphism analysis. *Appl. Environ. Microbiol.* 83:e00130-17. 10.1128/AEM.00130-17 28314730 PMC5440707

[B33] Opriessnig T. Hoffman L. J. Harris D. L. Gaul S. B. Halbur P. G. (2004). *Erysipelothrix rhusiopathiae*: genetic characterization of midwest US isolates and live commercial vaccines using pulsed-field gel electrophoresis. *J. Vet. Diagn. Invest.* 16 101–107. 10.1177/104063870401600202 15053359

[B34] Opriessnig T. Shen H. G. Bender J. S. Boehm J. R. Halbur P. G. (2013). *Erysipelothrix rhusiopathiae* isolates recovered from fish, a harbour seal (*Phoca vitulina*) and the marine environment are capable of inducing characteristic cutaneous lesions in pigs. *J. Comp. Pathol.* 148 365–372. 10.1016/j.jcpa.2012.08.004 23083834

[B35] Pomaranski E. K. Reichley S. R. Yanong R. Shelley J. Pouder D. B. Wolf J. C. (2017). Characterization of spaC-type *Erysipelothrix* sp. isolates causing systemic disease in ornamental fish. *J. Fish Dis.* 41 49–60. 10.1111/jfd.12673 28708262

[B36] Pomorska-Mól M. Markowska-Daniel I. Pejsak Z. (2012). Effect of age and maternally-derived antibody status on humoral and cellular immune responses to vaccination of pigs against *Erysipelothrix rhusiopathiae*. *Vet. J.* 194 128–130. 10.1016/j.tvjl.2012.03.009 22498786

[B37] Rice P. Longden I. Bleasby A. (2000). EMBOSS: the european molecular biology open software suite. *Trends Genet.* 16 276–277. 10.1016/s0168-9525(00)02024-2 10827456

[B38] Roy A. Kucukural A. Zhang Y. (2010). I-TASSER: a unified platform for automated protein structure and function prediction. *Nat. Protoc.* 5 725–738. 10.1038/nprot.2010.5 20360767 PMC2849174

[B39] Sawada T. Takahashi T. (1987). Cross protection of mice and swine inoculated with culture filtrate of attenuated *Erysipelothrix rhusiopathiae* and challenge exposed to strains of various serovars. *Am. J. Vet. Res.* 48 239–242. 3826862

[B40] Shen H. G. Bender J. S. Opriessnig T. (2010). Identification of surface protective antigen (spa) types in *Erysipelothrix* reference strains and diagnostic samples by spa multiplex real-time and conventional PCR assays. *J. Appl. Microbiol.* 109 1227–1233. 10.1111/j.1365-2672.2010.04746.x 20477888

[B41] Shi F. Ogawa Y. Sano A. Harada T. Hirota J. Eguchi M. (2013). Characterization and identification of a novel candidate vaccine protein through systematic analysis of extracellular proteins of *Erysipelothrix rhusiopathiae*. *Infect. Immun.* 81 4333–4340. 10.1128/IAI.00549-13 24019408 PMC3837990

[B42] Shimoji Y. (2000). Pathogenicity of *Erysipelothrix rhusiopathiae*: virulence factors and protective immunity. *Microbes Infect.* 2 965–972. 10.1016/s1286-4579(00)00397-x 10962280

[B43] Shimoji Y. Mori Y. Fischetti V. A. (1999). Immunological characterization of a protective antigen of *Erysipelothrix rhusiopathiae*: identification of the region responsible for protective immunity. *Infect. Immun.* 67 1646–1651. 10084998 10.1128/iai.67.4.1646-1651.1999 PMC96508

[B44] Shimoji Y. Ogawa Y. Osaki M. Kabeya H. Maruyama S. Mikami T. (2003). Adhesive surface proteins of *Erysipelothrix rhusiopathiae* bind to polystyrene, fibronectin, and type I and IV collagens. *J. Bacteriol.* 185 2739–2748. 10.1128/jb.185.9.2739-2748.2003 12700253 PMC154401

[B45] Shimoji Y. Ogawa Y. Tsukio M. Shiraiwa K. Nishikawa S. Eguchi M. (2019). Genome-wide identification of virulence genes in *Erysipelothrix rhusiopathiae*: use of a mutant deficient in a tagF homolog as a safe oral vaccine against swine erysipelas. *Infect. Immun.* 18:e673-19. 10.1128/IAI.00673-19 31548316 PMC6867862

[B46] Shiraiwa K. Ogawa Y. Nishikawa S. Eguchi M. Shimoji Y. (2018). Identification of serovar 1a, 1b, 2, and 5 strains of *Erysipelothrix rhusiopathiae* by a conventional gel-based PCR. *Vet. Microbiol.* 225 101–104. 10.1016/j.vetmic.2018.09.014 30322520

[B47] Sweredoski M. J. Baldi P. (2008). PEPITO: improved discontinuous B-cell epitope prediction using multiple distance thresholds and half sphere exposure. *Bioinformatics* 24 1459–1460. 10.1093/bioinformatics/btn199 18443018

[B48] Takahashi T. Fujisawa T. Umeno A. Kozasa T. Yamamoto K. Sawada T. (2008). A taxonomic study on *Erysipelothrix* by DNA-DNA hybridization experiments with numerous strains isolated from extensive origins. *Microbiol. Immunol.* 52 469–478. 10.1111/j.1348-0421.2008.00061.x 18822080

[B49] Takahashi T. Nagamine N. Kijima M. Suzuki S. Takagi M. Tamura Y. (1996). Serovars of *Erysipelothrix* strains isolated from pigs affected with erysipelas in Japan. *J. Vet. Med. Sci.* 58 587–589. 10.1292/jvms.58.587 8811634

[B50] Takahashi T. Takagi M. Sawada T. Seto K. (1984). Cross protection in mice and swine immunized with live erysipelas vaccine to challenge exposure with strains of *Erysipelothrix rhusiopathiae* of various serotypes. *Am. J. Vet. Res.* 45 2115–2118. 6497110

[B51] Tang H.-B. Xie J. Wang L. Liu F. Wu J. (2016). Complete genome sequence of *Erysipelothrix rhusiopathiae* strain GXBY-1 isolated from acute swine erysipelas outbreaks in south China. *Genom Data* 8 70–71. 10.1016/j.gdata.2016.04.006 27222802 PMC4856827

[B52] To H. Nagai S. (2007). Genetic and antigenic diversity of the surface protective antigen proteins of *Erysipelothrix rhusiopathiae*. *Clin. Vaccine Immunol.* 14 813–820. 10.1128/CVI.00099-07 17475766 PMC1951066

[B53] To H. Sato H. Tazumi A. Tsutsumi N. Nagai S. Iwata A. (2012). Characterization of *Erysipelothrix rhusiopathiae* strains isolated from recent swine erysipelas outbreaks in Japan. *J. Vet. Med. Sci.* 74 949–953. 10.1292/jvms.11-0533 22446396

[B54] To H. Someno S. Nagai S. Koyama T. Nagano T. (2010). Immunization with truncated recombinant protein SpaC of *Erysipelothrix rhusiopathiae* strain 715 serovar 18 confers protective immunity against challenge with various serovars. *Clin. Vaccine Immunol.* 17 1991–1997. 10.1128/CVI.00213-10 20926696 PMC3008202

[B55] Uchiyama M. Shimazaki Y. Isshiki Y. Kojima A. Hirano F. Yamamoto K. (2017). Pathogenic characterization of *Erysipelothrix rhusiopathiae* Met-203 type SpaA strains from chronic and subacute swine erysipelas in Japan. *J. Vet. Med. Sci.* 79 18–21. 10.1292/jvms.16-0164 27773881 PMC5289231

[B56] Uchiyama M. Yamamoto K. Ochiai M. Yamamoto T. Hirano F. Imamura S. (2014). Prevalence of Met-203 type spaA variant in *Erysipelothrix rhusiopathiae* isolates and the efficacy of swine erysipelas vaccines in Japan. *Biologicals* 42 109–113. 10.1016/j.biologicals.2013.12.002 24405986

[B57] Wood R. L. (1979). Specificity in response of vaccinated swine and mice to challenge exposure with strains of *Erysipelothrix rhusiopathiae* of various serotypes. *Am. J. Vet. Res.* 40 795–801. 112891

[B58] Wood R. L. Booth G. D. Cutlip R. C. (1981). Susceptibility of vaccinated swine and mice to generalized infection with specific serotypes of *Erysipelothrix rhusiopathiae*. *Am. J. Vet. Res.* 42 608–614. 6174056

[B59] Wood R. L. Harrington R. Jr. (1978). Serotypes of *Erysipelothrix rhusiopathiae* isolated from swine and from soil and manure of swine pens in the United States. *Am. J. Vet. Res.* 39 1833–1840. 736341

[B60] Zhang Y. Skolnick J. (2005). TM-align: a protein structure alignment algorithm based on the TM-score. *Nucleic Acids Res.* 33 2302–2309. 10.1093/nar/gki524 15849316 PMC1084323

[B61] Zhu W. Cai C. Wang Y. Li J. Wu C. Kang C. (2017a). Characterization of roles of SpaA in *Erysipelothrix rhusiopathiae* adhesion to porcine endothelial cells. *Microb. Pathog.* 113 176–180. 10.1016/j.micpath.2017.10.020 29038054

[B62] Zhu W. Wang Y. Cai C. Li J. Chao W. Jin M. (2017b). *Erysipelothrix rhusiopathiae* recruit host plasminogen via the major protective antigen SpaA. *FEMS Microbiol. Lett.* 364:fnx036 10.1093/femsle/fnx036 28201685

[B63] Zhu W. Wu C. Kang C. Cai C. Wang Y. Li J. (2018). Evaluation of the protective efficacy of four newly identified surface proteins of *Erysipelothrix rhusiopathiae*. *Vaccine* 36 8079–8083. 10.1016/j.vaccine.2018.10.071 30446176

[B64] Zhu W. Zhang Q. Li J. Wei Y. Cai C. Liu L. (2017c). Glyceraldehyde-3-phosphate dehydrogenase acts as an adhesin in *Erysipelothrix rhusiopathiae* adhesion to porcine endothelial cells and as a receptor in recruitment of host fibronectin and plasminogen. *Vet. Res.* 48:16. 10.1186/s13567-017-0421-x 28327178 PMC5360030

[B65] Zou Y. Zhu X. Muhammad H. M. Jiang P. Li Y. (2015). Characterization of *Erysipelothrix rhusiopathiae* strains isolated from acute swine erysipelas outbreaks in Eastern China. *J. Vet. Med. Sci.* 77 653–660. 10.1292/jvms.14-0589 25649849 PMC4488401

