# Supplementary material for: Genomic and Immunogenic Protein Diversity of Erysipelothrix rhusiopathiae Isolated From Pigs in Great Britain: Implications for Vaccine Protection
Source: Front Microbiol. 2020 Mar 13;11:418. doi: 10.3389/fmicb.2020.00418 (PMC7083082; doi:10.3389/fmicb.2020.00418)
Supplement: Supplementary file 1 [file Data_Sheet_1.DOCX]

Supplementary File 1


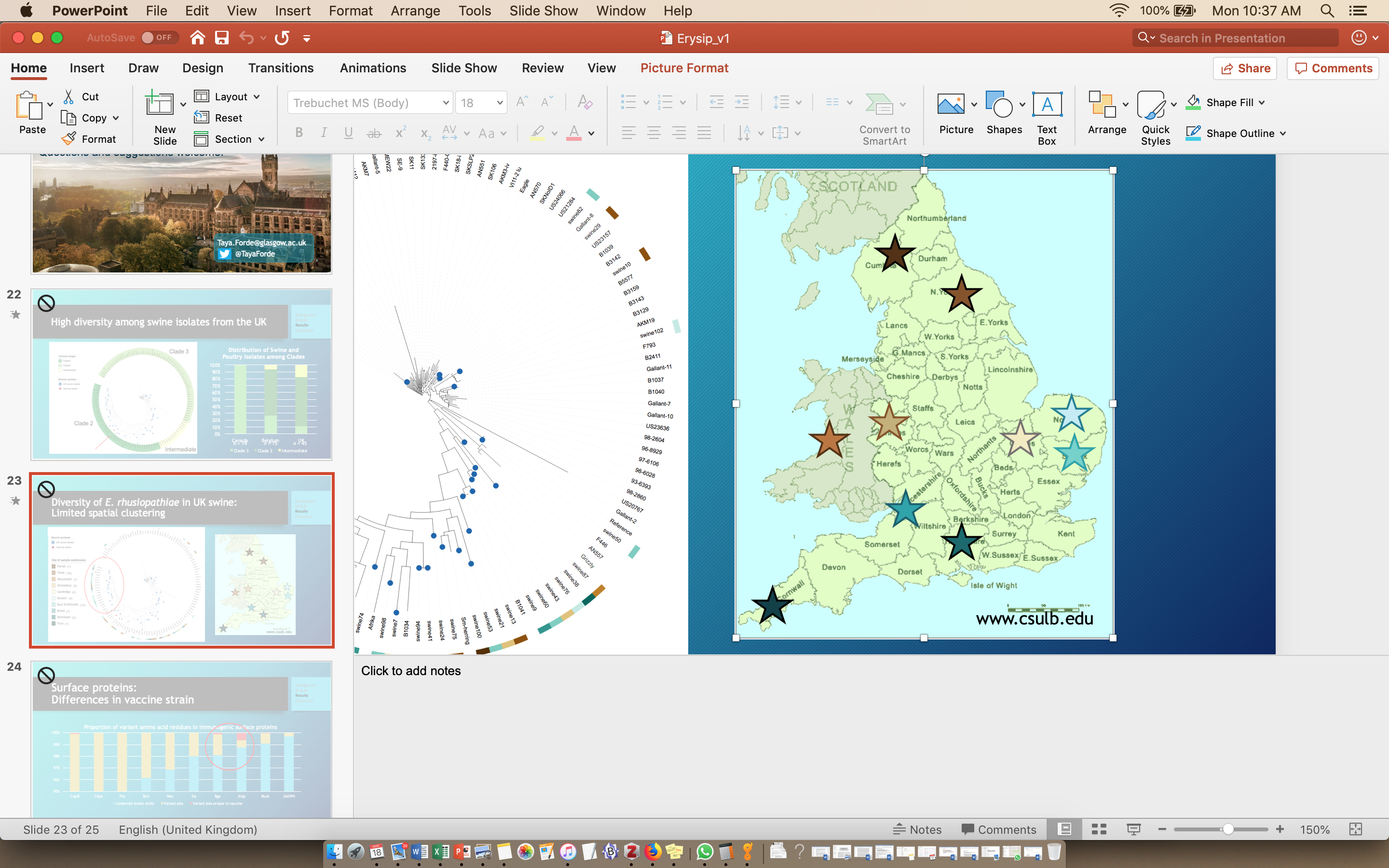


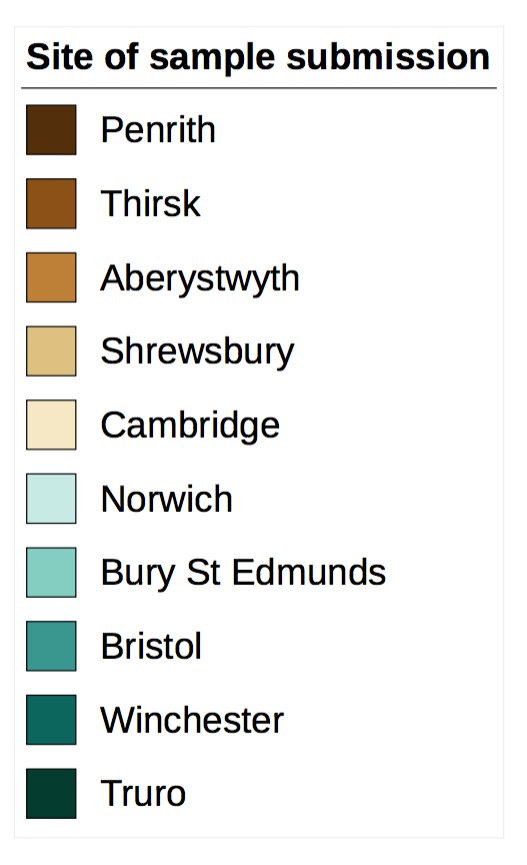


(1)

(10)

(1)

(2)

(2)

(3)

(14)

(7)

(3)

(1)

**Supplementary Figure 1.** **Locations of Veterinary Investigation Centres in England and Wales that provided isolates that were included in this study.** Numbers in parentheses show the number of isolates per centre.


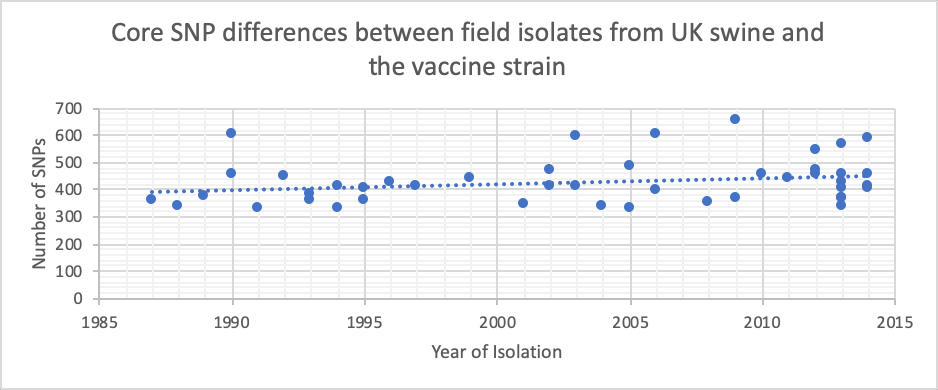


**Supplementary Figure 2.** **Core SNP differences between the Porcillis Ery® vaccine strain and field strains isolated from UK swine between 1987 and 2014.** Each individual point represents a single isolate.

**

**Supplementary Figure 3.** Homoplasy in variants of SpaA group 2. This phylogenetic tree is the same as Figure 3 (i.e. based on core single nucleotide polymorphisms (SNPs), estimated using the Nullarbor pipeline). British pig isolates are represented by blue circles at branch tips, while the vaccine strain is represented by a red star. SpaA group is shown by the yellow/red color strip. Variants of SpaA Group 2 have one or two variant amino acid positions as indicated by the dashed numbers.

**Supplementary Figure 4.** Assessment of changes in surface proteins over time. Proportion of variants for the different surface proteins before 2008 and from 2008 onwards. Type 1 is the variant that includes the vaccine strain (see Supp File 2).

The Fisher exact test statistic value is 0.0698. The result is not significant at p < .05.

The Fisher exact test statistic value is 0.1144. The result is not significant at p < .05.
