# Supplementary material for: Genomic and Immunogenic Protein Diversity of Erysipelothrix rhusiopathiae Isolated From Pigs in Great Britain: Implications for Vaccine Protection
Source: Front Microbiol. 2020 Mar 13;11:418. doi: 10.3389/fmicb.2020.00418 (PMC7083082; doi:10.3389/fmicb.2020.00418)
Supplement: Supplementary file 2 [file Data_Sheet_2.DOCX]

Supplementary File 2

Phylogenies of immunogenic surface proteins (mid-point rooted), based on amino acid sequences. Phylogenies are not shown for GADPH, Atsp, or Bml because of the limited variability (Supp Table 5), nor is one shown for cwpA because of the high degree of variability in the presence/absence of this protein sequence. These groupings form the basis of the clusters shown in Fig 3, and in Supp Table 1. Lineages highlighted in red (designated by the number 1) are those in which the vaccine strain is present.

# rspA


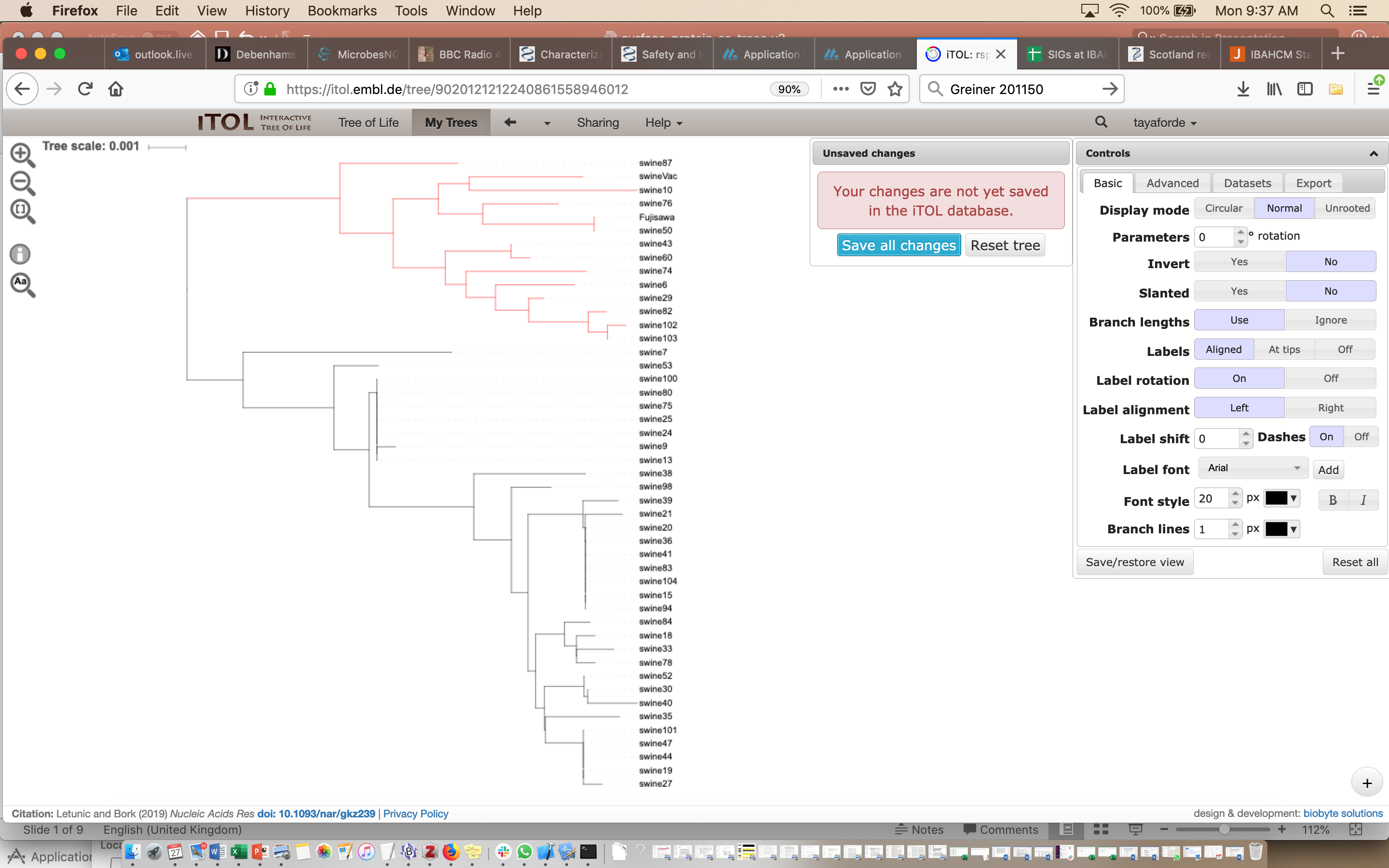


1

2

# cbpB


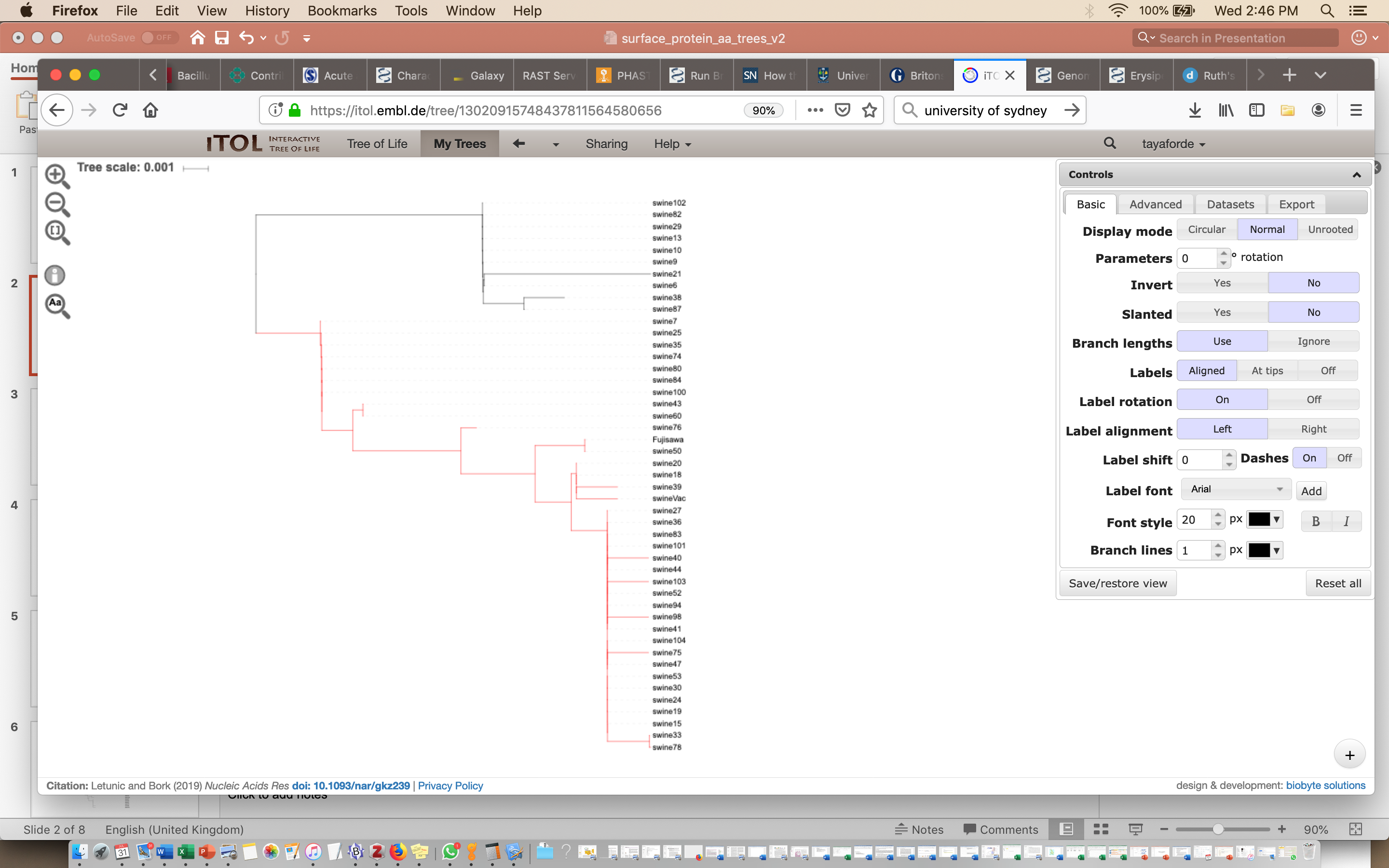


1

2

# Da


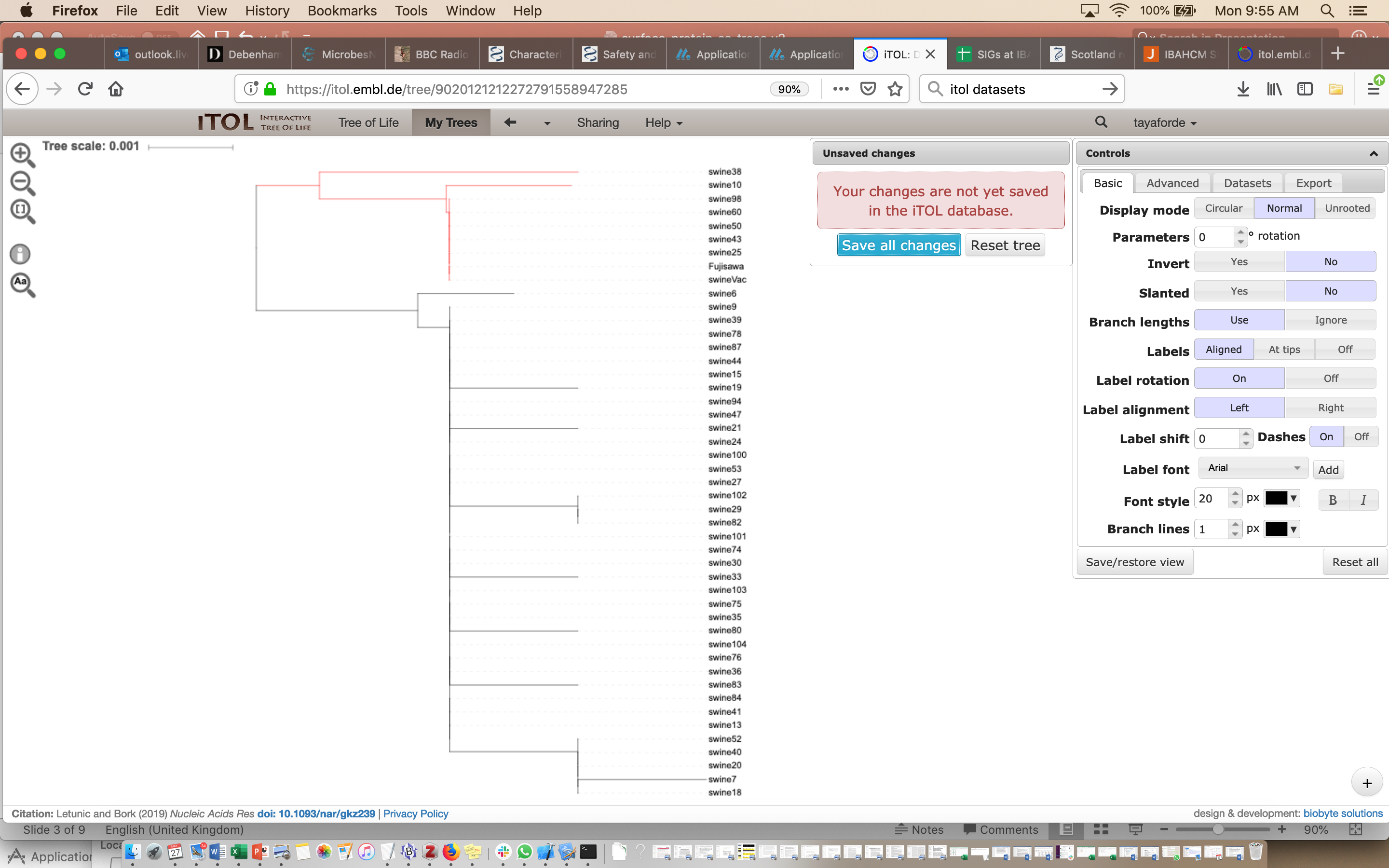


1

2

# cbpA


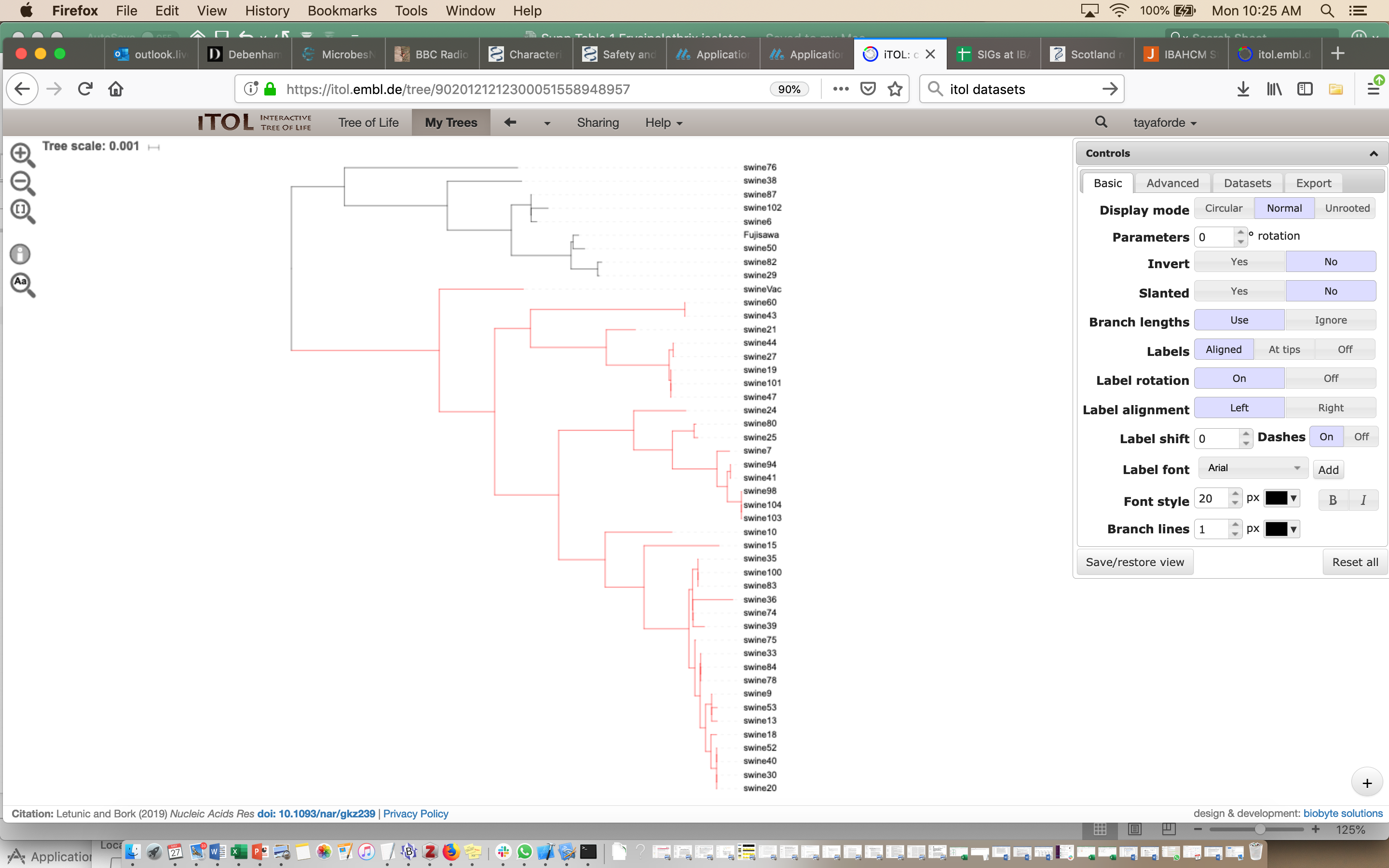


1

2

# Plp


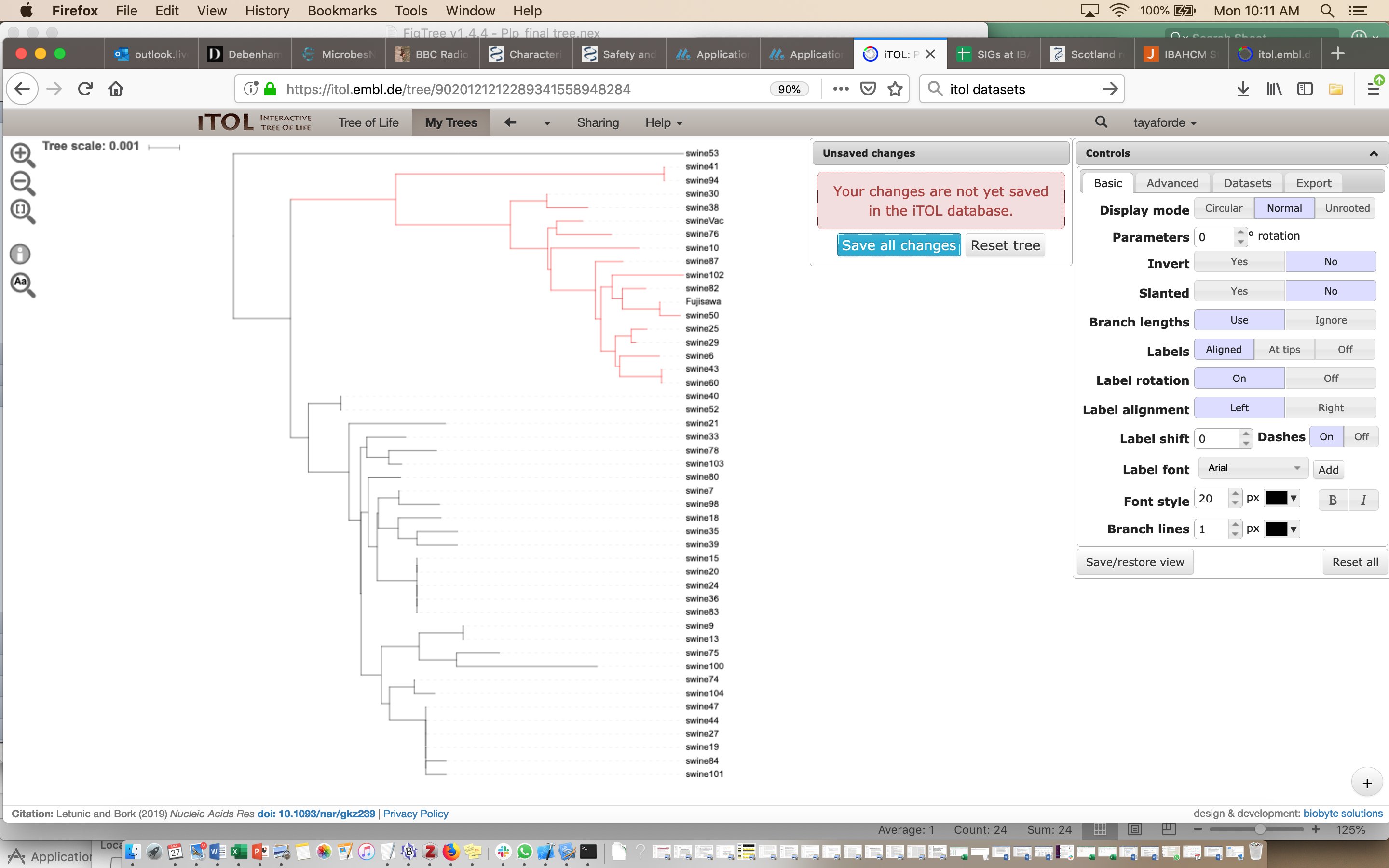


1

2

3

# Neu


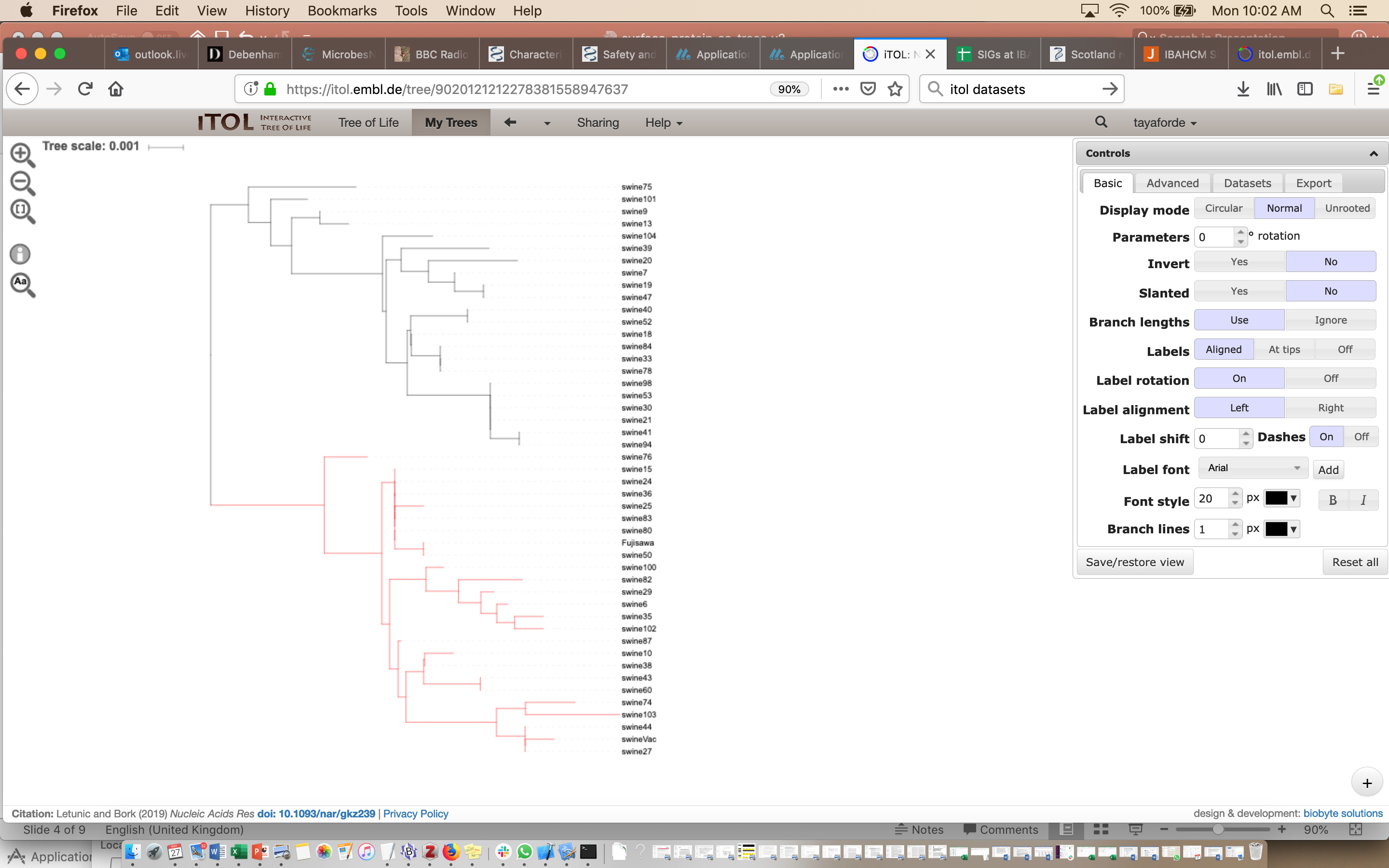


1

2

# Bga


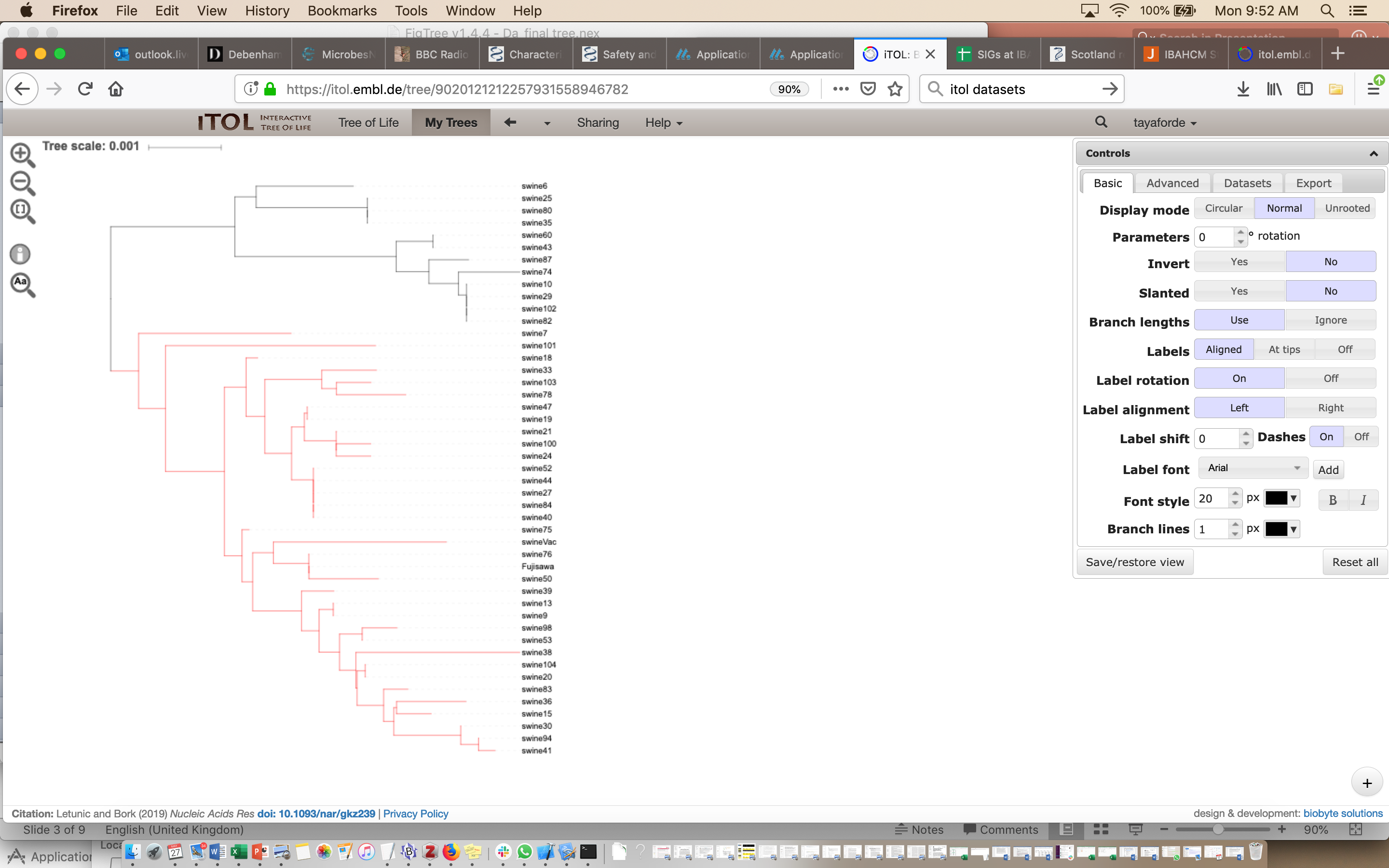


2

1

# cwpB


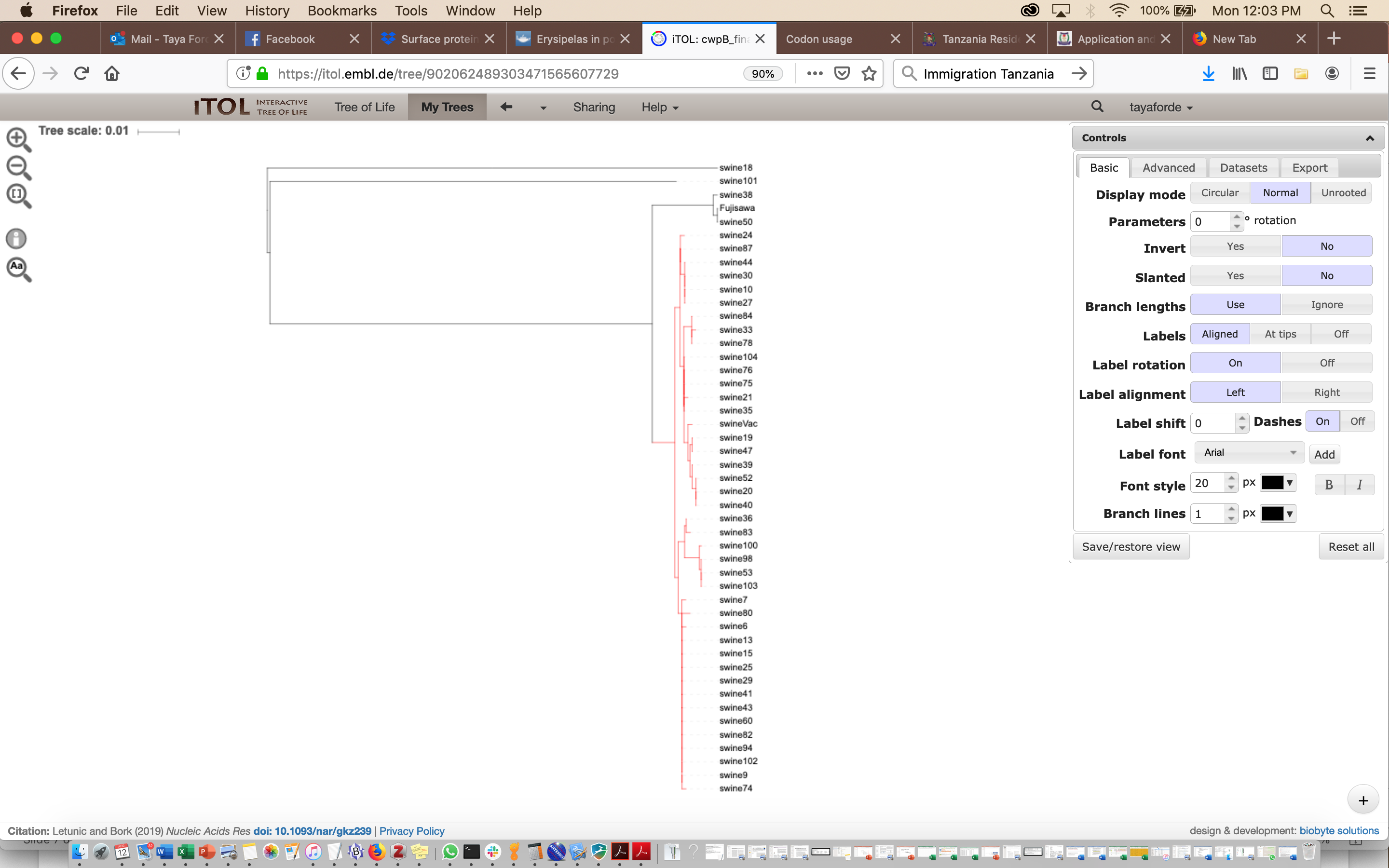


1

2

3

4
