# Supplementary material for: Genomic and Immunogenic Protein Diversity of Erysipelothrix rhusiopathiae Isolated From Pigs in Great Britain: Implications for Vaccine Protection
Source: Front Microbiol. 2020 Mar 13;11:418. doi: 10.3389/fmicb.2020.00418 (PMC7083082; doi:10.3389/fmicb.2020.00418)
Supplement: Supplementary file 3 [file Data_Sheet_3.docx]

Supplementary File 3

# Details of SpaA variability

## Immunoprotective Domain

Prior to this study, different groups of SpaA sequences were described based on differences in the amino acid (AA) sequence of the N-terminal hypervariable region (Uchiyama et al., 2014; Janßen et al., 2015). Uchiyama et al. initially developed a classification scheme based on 3 AA substitutions that clustered isolates into distinct groups. This scheme was expanded upon by Janßen et al., who divided SpaA sequences into 5 different groups, based on 8 variable AA sites.

Group 1 – as referred to by both this study and by Janßen et al., 2015 – has 5 AA positions within this domain that differ from the Fujisawa reference genome. It should be noted that the AA change at Position 303 in SpaA Group 1 is to Glutamic Acid (E) as opposed to Glutamine (Q) as was previously suggested (Janßen et al., 2015). These authors, as well as Uchiyama et al., 2014 (Uchiyama et al., 2014), also mentioned the AA difference at position 257 (Ile instead of Fujisawa’s Leu), but in this study, that AA was found to be unique to Fujisawa among all 343 SpaA sequences investigated, and was therefore not discriminatory within this study. However, this variant appears to be more common among swine in Japan (Ogawa et al., 2017). Another variable site previously noted among swine in Japan (242 E🡪D) (Ogawa et al., 2017; Uchiyama et al., 2017) was not found among our collection.

A subgroup of isolates with the Group 1 SpaA sequence (n= 8) had an additional variant at AA position 109 (N🡪H). This included the sequenced vaccine strain. There were three additional “variants” of Group 1 SpaA sequences, wherein one isolate had a different allele at one of the determinant AA positions (Supp Table 3). One of these was an alternate allele at AA101 (N🡪I) in isolate KR606142. The other two isolates, KR606245 and 20767, had the reference allele at AA 303 and 195, respectively.

As shown in Figure 4, the other AA variants of SpaA cluster phylogenetically, and can be seen to be minor variants of a common type, which we refer to as Group 2. The way in which they map to the previous classification schemes is detailed in Supp Table 3. None of the SpaA sequences we examined were identified as belonging to Janßen et al.’s previous ‘Group 4’ (Janßen et al., 2015).

## Proline-rich region

Two variable sites (AA positions 426 and 435) were found in the proline-rich hydrophobic area (positions 414-447) which discriminate among isolates. The variants at these positions in Fujisawa, 426-E and 435-L, are present in 74 isolates, while the other alleles, 426-K and 435-P, were found in 266 isolates (Supp Table 3). Only 3 isolates didn’t follow this pattern, and had the variants KL. These discriminatory variants were not included in the typing scheme because this region is not thought to contribute to the immunogenicity of the protein (Imada et al., 1999; To et al., 2010).

## Signal sequence

Few variants were found within the N-terminal signal sequence of 29 AAs of the SpaA protein, which is in keeping with earlier findings (To and Nagai, 2007; Janßen et al., 2015), and is suggestive of a high degree of selective pressure. It has been proposed that this sequence is associated with the secretion mechanism for this protein, and is conserved across Spa types (To and Nagai, 2007). Only five AA differences were found, and each was limited to a single isolate (Supp Table 4). These were at position 3 (K🡪N), position 9 (H🡪Y), position 10 (K🡪E), position 15 (S🡪T), and position 23 (P🡪S). The start codon varied for 3 isolates from GenBank, which had a leucine (L) instead of methionine (M).

## Repeat domain

The C-terminal repeat domain of SpaA, comprised of tandem repeats of 20 AAs with a GW module, is structurally similar among Spa types (To and Nagai, 2007), and indeed highly conserved among choline binding proteins of Gram positive bacteria (Makino et al., 1998; Jedrzejas, 2001). It is believed that this motif is what allows SpaA to bind to phosphorylcholine of the of *E. rhusiopathiae* capsule (Harada et al., 2014), facilitating binding to host endothelial cells. Janßen et al. found substantial variability in the number of tandem repeats among the *E. rhusiopathiae* strains they examined (7-13), although the majority of strains (~90%) had 9 repeats (Janßen et al., 2015). As these authors reflected, whether the number of repeats has a functional relevance, e.g. in the ability of the protein to bind to the bacterial cell surface, remains to be determined. Given the difficulty in accurately reconstructing repetitive sequences from shotgun sequence data, we did not investigate this component of the SpaA protein in this study.

**References**

Harada, T., Ogawa, Y., Eguchi, M., Shi, F., Sato, M., Uchida, K., et al. (2014). Phosphorylcholine and SpaA, a choline-binding protein, are involved in the adherence of Erysipelothrix rhusiopathiae to porcine endothelial cells, but this adherence is not mediated by the PAF receptor. *Vet. Microbiol.* 172, 216–222. doi:10.1016/j.vetmic.2014.04.012.

Imada, Y., Goji, N., Ishikawa, H., Kishima, M., and Sekizaki, T. (1999). Truncated surface protective antigen (SpaA) of Erysipelothrix rhusiopathiae serotype 1a elicits protection against challenge with serotypes 1a and 2b in pigs. *Infect. Immun.* 67, 4376–4382.

Janßen, T., Voss, M., Kühl, M., Semmler, T., Philipp, H.-C., and Ewers, C. (2015). A combinational approach of multilocus sequence typing and other molecular typing methods in unravelling the epidemiology of Erysipelothrix rhusiopathiae strains from poultry and mammals. *Veterinary Research* 46, 84. doi:10.1186/s13567-015-0216-x.

Jedrzejas, M. J. (2001). Pneumococcal virulence factors: structure and function. *Microbiol. Mol. Biol. Rev.* 65, 187-207 ; first page, table of contents. doi:10.1128/MMBR.65.2.187-207.2001.

Makino, S., Yamamoto, K., Murakami, S., Shirahata, T., Uemura, K., Sawada, T., et al. (1998). Properties of repeat domain found in a novel protective antigen, SpaA, of Erysipelothrix rhusiopathiae. *Microb. Pathog.* 25, 101–109.

Ogawa, Y., Shiraiwa, K., Ogura, Y., Ooka, T., Nishikawa, S., Eguchi, M., et al. (2017). Clonal Lineages of Erysipelothrix rhusiopathiae Responsible for Acute Swine Erysipelas in Japan Identified by Using Genome-Wide Single-Nucleotide Polymorphism Analysis. *Appl. Environ. Microbiol.* 83. doi:10.1128/AEM.00130-17.

To, H., and Nagai, S. (2007). Genetic and antigenic diversity of the surface protective antigen proteins of Erysipelothrix rhusiopathiae. *Clin. Vaccine Immunol.* 14, 813–820. doi:10.1128/CVI.00099-07.

To, H., Someno, S., Nagai, S., Koyama, T., and Nagano, T. (2010). Immunization with truncated recombinant protein SpaC of Erysipelothrix rhusiopathiae strain 715 serovar 18 confers protective immunity against challenge with various serovars. *Clin. Vaccine Immunol.* 17, 1991–1997. doi:10.1128/CVI.00213-10.

Uchiyama, M., Shimazaki, Y., Isshiki, Y., Kojima, A., Hirano, F., Yamamoto, K., et al. (2017). Pathogenic characterization of Erysipelothrix rhusiopathiae Met-203 type SpaA strains from chronic and subacute swine erysipelas in Japan. *J. Vet. Med. Sci.* 79, 18–21. doi:10.1292/jvms.16-0164.

Uchiyama, M., Yamamoto, K., Ochiai, M., Yamamoto, T., Hirano, F., Imamura, S., et al. (2014). Prevalence of Met-203 type spaA variant in Erysipelothrix rhusiopathiae isolates and the efficacy of swine erysipelas vaccines in Japan. *Biologicals* 42, 109–113. doi:10.1016/j.biologicals.2013.12.002.
